# Using Genomic Structural Equation Modeling to Partition the Genetic Covariance Between Birthweight and Cardiometabolic Risk Factors into Maternal and Offspring Components in the Norwegian HUNT Study

**DOI:** 10.1007/s10519-022-10116-9

**Published:** 2022-11-02

**Authors:** Gunn-Helen Moen, Michel Nivard, Laxmi Bhatta, Nicole M Warrington, Cristen Willer, Bjørn Olav Åsvold, Ben Brumpton, David M. Evans

**Affiliations:** 1 Institute of Clinical Medicine, Faculty of Medicine, University of Oslo, Oslo, Norway; 2 Institute of Molecular Biosciences, The University of Queensland, Brisbane, Australia; 3 Department of Public Health and Nursing, K.G. Jebsen Center for Genetic Epidemiology, NTNU, Norwegian University of Science and Technology, Trondheim, Norway; 4 Population Health Science, Bristol Medical School, University of Bristol, Bristol, UK; 5 The University of Queensland Diamantina Institute, The University of Queensland, 4102 Woolloongabba, QLD Australia; 6 Department of Biological Psychology, Vrije Universiteit Amsterdam, Amsterdam, The Netherlands; 7 Amsterdam Public Health Research Institute, Amsterdam University Medical Centers, Amsterdam, The Netherlands; 8 Department of Computational Medicine and Bioinformatics, University of Michigan, Ann Arbor, USA; 9 Department of Internal Medicine, University of Michigan, Ann Arbor, MI US; 10 Department of Human Genetics, University of Michigan, Ann Arbor, USA; 11 Department of Endocrinology, Clinic of Medicine, St. Olavs Hospital, Trondheim University Hospital, Trondheim, Norway; 12 Department of Public Health and Nursing, HUNT Research Centre, NTNU, Norwegian University of Science and Technology, Trondheim, Norway; 13 Clinic of Medicine, St. Olavs Hospital, Trondheim University Hospital, Trondheim, Norway; 14 Medical Research Council Integrative Epidemiology Unit, University of Bristol, Bristol, UK

**Keywords:** Birthweight, Maternal genetic effect, Offspring genetic effect, Genomic SEM, Developmental Origin of Health and Disease

## Abstract

The Barker Hypothesis posits that adverse intrauterine environments result in fetal growth restriction and increased risk of cardiometabolic disease through developmental compensations. Here we introduce a new statistical model using the genomic SEM software that is capable of simultaneously partitioning the genetic covariation between birthweight and cardiometabolic traits into maternally mediated and offspring mediated contributions. We model the covariance between birthweight and later life outcomes, such as blood pressure, non-fasting glucose, blood lipids and body mass index in the Norwegian HUNT study, consisting of 15,261 mother-eldest offspring pairs with genetic and phenotypic data. Application of this model showed some evidence for maternally mediated effects of systolic blood pressure on offspring birthweight, and pleiotropy between birthweight and non-fasting glucose mediated through the offspring genome. This underscores the importance of genetic links between birthweight and cardiometabolic phenotypes and offer alternative explanations to environmentally based hypotheses for the phenotypic correlation between these variables.

**Supplementary Information:**

The online version contains supplementary material available at 10.1007/s10519-022-10116-9.

## Introduction

There is a robust and well-documented relationship between lower birthweight and higher risk of cardiometabolic diseases like type 2 diabetes (T2D) and cardiovascular disease in later life. The Barker Hypothesis posits that adverse intrauterine environments result in fetal growth restriction and increased future risk of cardiometabolic disease through developmental compensations [1]. Evidence in favour of this theory has primarily come from observational epidemiological studies (which are susceptible to confounding, bias and reverse causality) [2–8]. However, because randomized controlled trials (RCTs) cannot realistically be performed in this context, definitive proof of the hypothesis in humans has been lacking.

In late 2016, the Early Growth Genetics (EGG) Consortium published a GWAS of birthweight using the UK Biobank (UKBB) and several birth cohorts from around the world [9]. This study increased the number of known loci for birthweight from 7 to 60 and provided important insights into the aetiology of the trait including the involvement of several type 2 diabetes and blood pressure associated variants. However, one of the most striking findings from the study was the demonstration that the well-known negative phenotypic correlation between birthweight and future risk of cardiometabolic disease [1] was in fact primarily mediated by genetic factors [9]. This finding is important because many theories concerning the origin of this relationship, like the Barker Hypothesis, have primarily focused on the role of environmental factors (i.e. growth restriction *in utero* as a consequence of nutritional deficiency causes long term developmental compensations that result in increased risk of cardiometabolic disease in later life), whereas the EGG study confirmed a major role for genetics in the genesis of this relationship.

Despite these surprising findings, the Horokoshi et al. (2016) [9] results are not necessarily inconsistent with environmental based hypotheses like Barker, since genetic correlations between birthweight and cardiometabolic disease could also arise through the maternal genome (i.e. which in turn influences the *in utero* environment). Indeed, in order to properly understand the meaning of the Horokoshi et al. (2016) [9] results, it is necessary to devise methods to partition genetic effects on birthweight (and cardiometabolic phenotypes) into maternal and offspring sources of variation.

Two years later, Warrington et al. published one such method based on structural equation modelling (SEM) [10] and applied this method to the analysis of own birthweight and offspring birthweight from > 320,000 individuals and > 230,000 mothers from the UK Biobank and EGG Consortium [11]. The authors then used these partitioned estimates of maternal and offspring genetic effects on birthweight in an LD score regression analysis [12, 13] to estimate the genetic correlation between own/offspring birthweight and cardiometabolic traits and diseases. Interestingly, the authors found evidence for a positive genetic correlation between many glucose-related parameters (e.g. fasting glucose, fasting insulin etc.) and maternal effects on birthweight, and a negative genetic correlation between glycemic parameters and offspring effects on birthweight [11]. These results are more consistent with a Fetal Insulin Hypothesis model of the relationship between birthweight and cardiometabolic disease, which posits that the same genetic factors that alter intrauterine growth also affect future risk of disease [14] (i.e. diabetes risk alleles in the mother result in higher levels of circulating glucose tending to increase offspring birthweight, whereas many of the same loci in the fetus decrease sensitivity to insulin, tending to decrease offspring birthweight, and predisposing the child to T2D in later life), than a Barker type model.

**Fig. 1:**
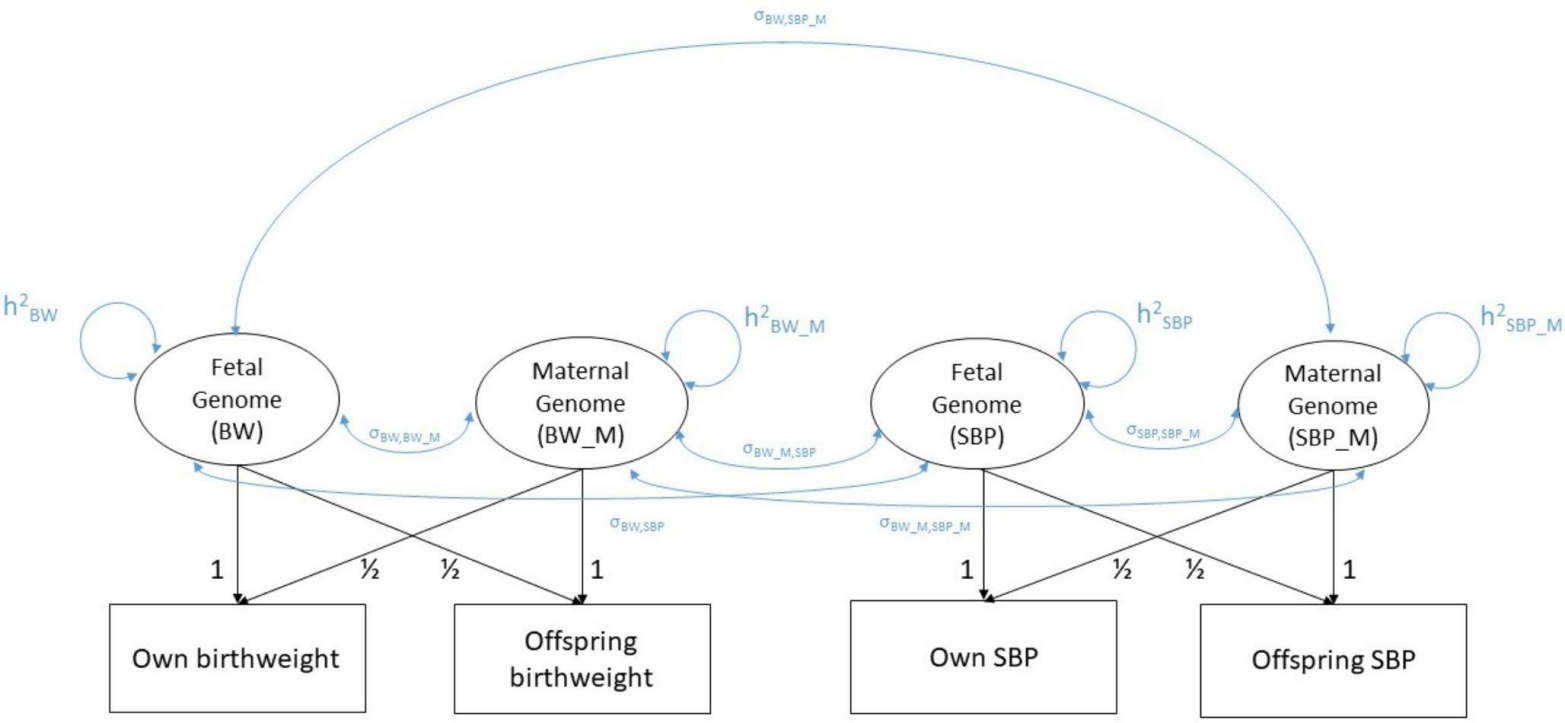
**Genomic SEM model.** Summary results statistics from two birthweight GWAS (BW) and two later-life trait GWAS (squares), in this case systolic blood pressure (SBP), are modelled in terms of latent variables representing the fetal genome and the maternal genome (circles). The lower part of this model reflects simple biometrical genetics principles (i.e. the fact that offspring and maternal genome are correlated 0.5) and consists of path coefficients fixed to the value of one or one half. The top half of the model consists of free parameters requiring estimation- four SNP heritabilities (one for each trait), and six genetic covariances between the different variables, representing commonalities in gene action across the maternal and fetal genomes. It is these covariance terms (particularly the covariances involving birthweight – cardiometabolic terms) that are of most interest for dissecting the negative correlation between birthweight and cardiometabolic risk factors. For example, a significant genetic covariance between the latent maternal BW and maternal SBP variables would be consistent with Barker Hypothesis type mechanisms

Nevertheless, whilst these results are interesting, they are not definitive. Although the authors were able to partition genetic effects on birthweight into maternal and offspring genetic effects, they did not do the same for cardiometabolic traits [11], whose GWAS summary results statistics may reflect a complicated mixture of maternal and offspring mediated components. In this manuscript, we introduce a new statistical model using the genomic SEM software [15], which is capable of simultaneously partitioning the genetic covariation between birthweight and cardiometabolic traits into maternally mediated and offspring mediated contributions (Fig. 1). We subsequently model the covariance between birthweight and later life outcomes, such as blood pressure, non-fasting glucose, blood lipids and body mass index (BMI) in the Trøndelag Health Study (HUNT), Norway [16]. The HUNT Study may be informative for investigating these relationships as it has a large number of mother-offspring pairs with genetic and phenotypic data and has offspring who are now are at an age where adverse values on cardiometabolic risk factors are beginning to become clinically apparent.

## Methods

### HUNT Study

The HUNT is a large population-based health study of the inhabitants of Trøndelag County in central Norway which commenced in 1984. A comprehensive description of the study population has been previously reported [16]. Approximately every 10 years the entire adult population of northern Trøndelag (~ 90,000 adults in 1995) is invited to attend a health survey which includes comprehensive questionnaires, an interview, clinical examination, and detailed phenotypic measurements (HUNT1 (1984 to 1986); HUNT2 (1995 to 1997); HUNT3 (2006 to 2008) and HUNT4 (2017 to 2019)). These surveys have high participation, with 89%, 69%, 54% and 54% of invited adults participating in HUNT1, 2, 3 and 4, respectively [16, 17]. Additional phenotypic information is collected by integrating national registers. Approximately 90% of participants from HUNT2 and HUNT3 were genotyped in 2015 [18], 2202 HUNT individuals had low-pass sequencing performed to improve imputation, and the genotype and phenotype data used in the subsequent analyses are exclusively from the HUNT2 and HUNT3 surveys.

### Genotyping, Quality Control and Imputation

Genotyping, quality control and imputation in the HUNT study have been described in detail elsewhere [19]. In short, DNA from 71,860 HUNT samples were genotyped using one of three different Illumina HumanCoreExome arrays (HumanCoreExome12 v1.0, HumanCoreExome12 v1.1 and UM HUNT Biobank v1.0). Genomic position, strand orientation and the reference allele of genotyped variants were determined by aligning their probe sequences against the human genome (Genome Reference Consortium Human genome build 37 and revised Cambridge Reference Sequence of the human mitochondrial DNA; http://genome.ucsc.edu) using BLAT [20]. Ancestry of all samples was inferred by projecting all genotyped samples into the space of the principal components of the Human Genome Diversity Project (HGDP) reference panel (938 unrelated individuals; downloaded from http://csg.sph.umich.edu/chaolong/LASER/) [21, 22], using PLINK v1.90 [23]. The resulting genotype data were phased using Eagle2 v2.3 [24]. Imputation was performed on the 69,716 samples of recent European ancestry using Minimac3 (v2.0.1, http://genome.sph.umich.edu/wiki/Minimac3) [25] with default settings (2.5 Mb reference based chunking with 500 kb windows) and a customized Haplotype Reference consortium release 1.1 (HRC v1.1) for autosomal variants, including 2202 HUNT low-pass genomes, and HRC v1.1 for chromosome X variants [26].

### Identifying Genotyped Mother-Offspring Pairs

Identification of genotyped mother-offspring pairs has previously been described in detail [27]. In short, plink files with genotyped SNPs underwent a second stage of cleaning. Any individuals whose inferred sex contradicted their reported gender (N = 348) as well as individuals showing high or low heterozygosity (+/- 5SD from the mean) (N = 412) were removed (760 individuals in total). In addition, variants with minor allele frequency < 0.005 or more than 5% missing rate were removed. Mother-offspring pairs were identified by kinship analysis using the KING software [28]. Only genotyped SNPs shared across the arrays on autosomal chromosomes were used for the analysis – a total of 257,488 SNPs. From the kinship analysis, 46,428 parent-offspring relationships were identified based on the recommended thresholds for relatedness implemented as part of this package [28]. Parent-offspring pairs and sibling pairs were distinguished according to their estimated probability of sharing zero alleles identical by decent (${\widehat{\pi }}_{0}$). This quantity was estimated using the KING software [28] which uses an inference threshold of ${\widehat{\pi }}_{0}$ < 0.1 to distinguish parent-offspring pairs from full sibling pairs who are expected to have 0.1 ${\le \widehat{\pi }}_{0}\le$ 0.365. Sex of the older individual was used to identify mother-offspring pairs, and any mother-offspring pair whose birth years were 15 years or less apart was removed from further analyses. A total of 26,057 mother-offspring pairs of European ancestry with genotype information passing QC were identified. There were several mothers with multiple offspring, so we selected the eldest offspring for the following analyses to ensure independence between observations (N = 15,261 pairs).

### Phenotypes

#### Birthweight

Individuals’ own birthweight and mothers reporting offspring birthweight were available for individuals in HUNT after linking with the Medical Birth Registry of Norway (MBRN) [29]. The registry commenced in 1967, when health authorities began reporting pregnancy-related data; therefore, birthweight measurements were only available for HUNT participants born in 1967 or later. The validity of birthweight information in the MBRN has previously been assessed as very good. One study reported high agreement between birthweight recorded in the MBRN and a selection of 786 HUNT women with matched hospital records, including 100% concordance between births classified as low (< 2500 g) or high birthweight (> 4500 g) [30]. Individuals in HUNT with own birthweight who reported in the registry to be a part of a multiple birth (210 twins and 4 triplets) were excluded from analyses. Additionally, we excluded individuals with congenital malformations (N = 317) and individuals where the birth was induced or performed by Cesarian-section (N = 2,488). The same exclusion criteria were applied to mothers (in the 26,057 mother-offspring pairs) with offspring birthweight recorded (N = 1,585 multiple births, N = 1,959 offspring with congenital malformation, N = 2,138 birth who were induced or performed by Cesarean-section). Additionally, the birthweight phenotypes were cleaned so that any birthweights under 1000 g were removed, as were any offspring born before 258 days of gestation or after 301 days of gestation. Lastly, birthweight was transformed to a Z-score before analysis.

#### Later Life Cardiometabolic Traits

During the four health surveys (HUNT1-4) [16] clinical examination, and detailed phenotypic measurements were performed on all participants. For all cardiometabolic risk factors in the offspring (BMI, systolic blood pressure (SBP), diastolic blood pressure (DBP), non-fasting glucose (Glucose), total cholesterol, high density lipoprotein (HDL) cholesterol, low density lipoprotein (LDL) cholesterol, and triglycerides), the values measured in HUNT3 were used if available. If the individuals were not a part of HUNT3, measurements from HUNT2 were used. Age at measurement was calculated to correspond with the age at the health survey chosen. Details regarding the phenotype measurement have been described in-depth previously [27]. In short, blood pressure was taken three times during the clinical examination, and SBP and DBP measurements were calculated as the average of the second and third measurements (second measurement was used if third not available). For the blood measurements, samples were taken from non-fasting participants. In HUNT3, participants’ total cholesterol was measured by enzymatic cholesterol esterase methodology; HDL cholesterol was measured by accelerator selective detergent methodology; triglycerides were measured by glycerol phosphate oxidase methodology; and glucose was measured by Hexokinase/G-6-PDH methodology (Abbott, Clinical Chemistry, USA). In HUNT2, participants’ total and HDL cholesterol and triglycerides were measured by applying enzymatic colorimetric cholesterol esterase methods (Boeheringer Mannheim, Mannheim, Germany) and glucose was measured by an enzymatic hexokinase method. Weight and height were measured in light clothes and BMI was calculated as weight (kilograms) divided by the squared value of height (in metres).

We adjusted the blood pressure measurements of individuals who self-reported using blood pressure lowering medication by adding 15 mmHg to their SBP and 10 mmHg to their DBP [31]. LDL cholesterol was calculated using the Friedewald formula [32]. All values more than 4 standard deviations from the mean were removed. If the variable was not normally distributed (HDL, triglycerides, BMI, and non-fasting glucose) the values were natural log transformed before removing outlying values.

### Phenotypic Correlations

Phenotypic correlations between individuals’ own birthweight and their eight later life traits were estimated using Pearson correlation coefficients. Individuals who had both birthweight and the later life trait available contributed to these analyses (N = 10,066). However, because the birth registry commenced in 1967, when health authorities began reporting pregnancy-related data, most of the individuals for whom both measures were available were young. We therefore also specifically calculated correlations in a subgroup of individuals who were 40 years or older (N = 512) on the basis that they were more likely to exhibit signs of cardiometabolic disease and therefore the magnitude of the negative correlation with birthweight might be larger.

### Genome-Wide Association Analysis

In the HUNT study, 16 GWAS were performed across eight cardiometabolic phenotypes (either individual’s own genotype and phenotype, or GWAS of maternal genotype and offspring phenotype; phenotypes included SBP, DBP, Glucose, BMI, LDL, HDL, triglycerides and total cholesterol) in addition to a GWAS of own birthweight and a GWAS of offspring birthweight. Linear mixed models were fit using BOLT-LMM [33] to account for the considerable cryptic relatedness within the HUNT population. Offspring sex and genotype batch were used as covariates, in addition to age at measurement for the later life phenotypes.

### GWAS Meta-Analysis of Birthweight

Because of the limited number of individuals in the HUNT study with birthweight information, we meta-analysed the HUNT GWAS of own birthweight (N = 10,066) and offspring birthweight (N = 23,688) with previously published GWAS summary results statistics from the Early Growth Genetics (EGG) Consortium (own birthweight N = 298,142, offspring birthweight N = 210,267) [11]. We combined the summary results statistics from the EGG meta-analysis with the HUNT summary results statistics using a fixed-effects meta-analysis using METAL [34] and performed the subsequent LD score regression analysis and genomic SEM analysis with these combined summary results statistics.

### LD Score Regression

To estimate the SNP-heritability and genetic correlation of the traits we used the CTG-VL platform [35]. All summary results statistics from the above mentioned GWAS were uploaded to the server and SNP-heritability and genetic correlations were calculated. CTG-VL uses pre-computed LD Scores from a European population provided by the original developers of LD score regression [36], using HapMap 3 SNPs with the MHC region excluded. It is important to realize that in contrast to the phenotypic correlations, estimates of the genetic correlation between birthweight and the later life traits effectively use data from all individuals with either birthweight or cardiometabolic information (or both measures). Thus, estimates of the genetic correlation/covariance between birthweight and later life traits will include many more individuals of advanced age than represented in the HUNT phenotypic correlation analyses.

### Genomic SEM

The Genomic SEM method [15] involves two stages. In the first stage, LD score regression methods using pre-computed LD Scores from a European population provided by the original developers of LD score regression [36] are applied to GWAS summary results statistics to estimate the genetic variance of each trait, and the genetic covariance between traits. In LD score regression, chi-square results for each SNP are regressed on their corresponding LD score (a measure of how many SNPs are in LD with the index SNP). The genetic variance of the trait is related to the slope from this regression [12]. Likewise, genetic covariances between traits can be estimated using bivariate LD score regression [36], where the product of chi-square terms for each SNP is regressed on the LD score for each SNP. In the second stage of genomic SEM, a user defined SEM is fit to the genetic covariance matrix and parameters and their standard errors are estimated.

The genomic structural equation model we used to partition genetic covariances into maternal and offspring components is displayed in Fig. 1 (using birthweight and systolic blood pressure as an exemplar). Results from two birthweight GWAS and two later-life trait GWAS (squares) were modelled in terms of latent maternal and offspring genetic variables (circles). The lower part of this model reflects simple biometrical genetics principles (i.e. the fact that offspring and maternal genome are correlated 0.5) and consists of path coefficients fixed to the value one or one half. The top half of the model consists of free parameters requiring estimation- four SNP heritabilities (one for each trait), and six genetic covariances between the variables, representing commonalities in genetic action across the maternal and offspring genomes. It is these covariance terms (particularly the covariances involving birthweight – cardiometabolic traits) that are of most interest for dissecting the purported negative correlation between birthweight and cardiometabolic risk factors. For example, a substantial negative genetic covariance between the latent genetic factor proxying “own birthweight” and the latent genetic factor proxying “own cardiometabolic trait” (σ_BW, SBP_) would emphasize the importance of genetic pleiotropy through an individual’s genome in the genesis of the correlation between birthweight and cardiometabolic risk factors. In contrast, a substantial genetic covariance between the latent genetic factor proxying “offspring birthweight” and the latent genetic factor proxying “offspring cardiometabolic risk factors” (σ_BW_M, SBP_M_), would point to the importance of maternal intrauterine influences and would be consistent with Barker Hypothesis type mechanisms. These different possibilities are described more in Table 1.

It is important to realise that fitting a complicated SEM like the one in Fig. 1 is necessary to obtain asymptotically unbiased estimates of SNP heritabilities and genetic correlations. The reason is that GWAS of perinatal (and potentially cardiometabolic) traits represent a complicated mixture of maternal and offspring genetic effects. Our SEM disentangles these effects from each contributing GWAS. In contrast, the model underlying LD Score regression makes no allowance for this complication, and so naïve use will lead to biased estimates of SNP heritability and genetic correlations containing an unknown mixture of maternal and fetal effects. Additionally, maternal (fetal) GWAS need to be corrected (either implicitly or explicitly) for the decrement in power that arises from using maternal (fetal) genotype as a proxy of offspring genotype to estimate direct fetal (indirect maternal) genetic effects- otherwise estimates of SNP heritability may be biased downwards (see de la Fuente et al. 2022 [37] and Supplementary Note 1 for a more detailed discussion of this point). In contrast, our model fitted using genomicSEM accounts for this concern and should produce asymptotically unbiased estimates of SNP heritability and genetic correlations arising from maternal and fetal genetic sources of variation. It is important to note that this partitioning of the estimated genetic covariance matrix into maternal and fetal components is a prerequisite to examining the potential existence of Barker hypothesis consistent mechanisms (i.e. which posit the existence of maternal effects on offspring birthweight and offspring cardiometabolic physiology) using genetic correlations and covariances.

**Table 1:** Explanation of genetic covariances in the genomic SEM

Latent factors contributing to genetic covariance	Putative Explanation
Fetal Genome (BW) – Maternal Genome (BW_M) (σ_BW,BW_M_)	Genetic variants that directly affect one’s own BW, when present in mothers, also affect their offspring’s BW.
Fetal Genome (SBP) – Maternal Genome (SBP_M) (σ_SBP,SBP_M_)	Genetic variants that directly affect one’s own SBP, when present in mothers, also affect their offspring’s SBP.
Fetal Genome (BW) – Fetal Genome (SBP) (σ_BW,SBP_)	Genetic pleiotropy (through the fetal genome). Genetic variants that directly affect one’s own BW also directly affect one’s own SBP.
Maternal Genome (BW_M) – Maternal Genome (SBP_M) (σ_BW_M,SBP_M_)	Genetic pleiotropy through the maternal genome. Genetic variants in the maternal genome affect both their offspring’s BW and their offspring’s SBP. **A significant component is consistent with the Barker Hypothesis**.
Fetal Genome (BW) – Maternal Genome (SBP_M) (σ_BW,SBP_M_)	Genetic variants that directly affect one’s own BW, when present in mothers, also affect their offspring’s SBP.
Fetal Genome (SBP)-Maternal Genome (BW_M) (σ_SBP,BW_M_)	Genetic variants that directly affect one’s own SBP, when present in mothers, also affect their offspring’s BW. Consistent with a causal effect of maternal SBP on offspring BW.

*We have chosen SBP as a model trait to make the explanations more concrete. BW: birthweight; BW_M offspring birthweight; SBP: Systolic Blood Pressure; SBP_M Offspring systolic blood pressure

Summary results statistics files from the GWASs described above were combined using genomic SEM [15]. The software was set to not exclude INDELs. Code for the model specified in the analysis is available in Supplementary Note 2.

## Results

Both the full HUNT cohort and offspring in the mother-offspring pairs consisted of 47.3% males (Table 2). Our GWAS analysis consisted of either the full HUNT study (N = 68,856 after genotype cleaning) for the own phenotype analysis or N = 15,261 mothers (genotype) with offspring phenotype for the offspring phenotype analysis. The exact sample size for each GWAS is shown in Table 2. Manhattan plots, and QQ plots for each of the 18 GWAS are shown in Supplementary Note 3, and genomic inflation factors along with univariate LD score regression intercepts are listed in Supplementary Table 1. To ensure that our GWAS results were consistent with previous GWAS in larger samples, we identified (up to) the top 10 genome-wide significant hits (after clumping r^2^ < 0.05, using a European reference panel [35]) for each trait and confirmed association in previous GWAS (Supplementary Table 2). Across all traits, SNPs that were known to be robustly associated with the trait of interest met the criteria for genome-wide significance in HUNT, consistent with the phenotyping and genotyping/imputation of the HUNT study being of high quality.

**Table 2:** Descriptive statistics of subset of study participants in the HUNT study used in the analyses

		Own phenotype			Offspring phenotype		
**Trait**	**Unit**	**N**	**Range**	**Mean (SD)**	**N**	**Range**	**Mean (SD)**
Birthweight*	grams	10,066	1390–5900	3554 (486)	23,688	1370–5900	3670 (491)
SBP	mmHg	68,781	60–238	137.5 (23.37)	15,186	70–218	128.4 (17.61)
DBP	mmHg	68,793	36–138	77.93 (13.54)	15,183	36–128	73.57 (12.00)
Glucose	mmol/L^a^	67,378	2.29–12.81	5.42 (1.21)	14,873	2.29–12.81	5.21 (1.19)
BMI	kg/m^2a^	68,595	14.89–50.40	26.58 (1.17)	15,192	15.80–49.40	26.31 (1.18)
LDL	mmol/L	67,909	0.65–9.38	3.95 (1.11)	14,926	0.82–9.38	3.63 (1.00)
HDL	mmol/L^a^	68,007	0.50–2.90	1.34 (0.35)	14,941	0.50–2.80	1.33 (0.33)
Triglycerides	mmol/L^a^	68,806	0.18–12.81	1.46 (1.70)	15,175	0.20–11.70	1.35 (1.72)
Total cholesterol	mmol/L	68,096	1.30–10.90	5.64 (1.19)	14,954	2.00-10.90	5.28 (1.08)
Age	Years	68,856	19.1-101.1	53.62 (17.41)	15,261	30.4–83.2	41.53 (13.38)

HUNT: Trøndelag Health Study; mmHg: millimeters of mercury; mmol/L: millimoles per litre; N: number of individuals; SD: standard deviation; SBP: Systolic Blood Pressure; DBP: Diastolic Blood Pressure; Glucose: Non-fasting Glucose; BMI: Body Mass Index; LDL: Low Density Lipoprotein; HDL: High Density Lipoprotein. ^a^Phenotype (natural) logarithm transformed in analyses but presented in untransformed units here. *Genome-wide association study of birthweight was performed on Z-score

The magnitude of the phenotypic correlation between individuals’ own birthweight and own later life traits (N = 10,066, average age of 30.54 years) was low (although p < 0.05 for all traits except SBP and triglycerides, Table 3). However, it is important to realise that these correlations primarily reflect the association between birthweight and cardiometabolic phenotypes in younger individuals, as information on birthweight was only available for HUNT participants born in 1967 or later. As adverse cardiometabolic changes typically clinically manifest in middle and old age, we were concerned that this ascertainment might have artificially depressed the magnitude of the phenotypic correlations presented in Table 3. We therefore stratified the HUNT sample on age and recalculated the phenotypic correlations in a subset of older individuals (N = 512; Age range: 40 to 41.1; Mean: 40.5 years). As expected, the magnitude of many of the correlations increased accordingly, with the data showing the expected negative correlations between birthweight and many cardiometabolic traits and a positive correlation with BMI, even though the correlations did not meet the criterion for statistical significance (p < 0.05) in the smaller sample (Table 3).

**Table 3:** Phenotypic correlation between birthweight and later life traits

	Individuals over 40 years old			All HUNT individuals		
**Trait**	**N**	**Correlation coefficient**	**p-value**	**N**	**Correlation coefficient**	**p-value**
SBP	511	-0.076	0.085	10,024	0.003	0.763
DBP	511	-0.031	0.480	10,024	-0.027	0.008
Glucose	495	-0.062	0.165	9,852	-0.032	0.002
BMI	511	0.053	0.227	10,032	0.051	3.43 × 10^− 7^
LDL	498	-0.026	0.562	9,872	-0.018	0.071
HDL	498	-0.039	0.380	9,882	-0.034	0.001
Triglycerides	510	-0.044	0.317	10,021	-0.002	0.849
Total Cholesterol	498	-0.048	0.288	9,885	-0.03	0.003

HUNT: Trøndelag Health Study; N: number of individuals; SBP: Systolic Blood Pressure; DBP: Diastolic Blood Pressure; Glucose: Non-fasting Glucose; BMI: Body Mass Index; LDL: Low Density Lipoprotein; HDL High Density Lipoprotein

There was strong evidence of heritability, estimated by LD score regression, for “own” phenotype, with SNP heritability estimates ranging between 3% (non-fasting glucose) and 20% (HDL, BMI) (Table 4). However, except for offspring birthweight, there was only limited evidence of SNP heritability for the “offspring” phenotypes. This result is surprising- given that maternal and offspring genotypes are correlated approximately 0.5, we would expect *a priori* that the maternal signal would proxy the offspring signal (even in the absence of genuine maternal effects) as we are not estimating maternal genetic effects conditional on offspring genetic effects in this analysis (in Supplementary Note 1 we show that approximately four times the sample size is required to detect a locus that acts directly through an individual’s own genome on their own phenotype by regressing offspring phenotype on maternal genotype with equivalent statistical power to regressing own phenotype on own genotype). Although some offspring phenotypes (BMI, total cholesterol and triglycerides) had significant evidence of heritability (p < 0.05), the Z scores for the test of heritability did not exceed Z > 4. The authors of LD score regression suggest that a Z score of 4 for SNP-heritability is a minimum threshold to obtain reliable estimates of genetic correlations, and consequently suggests that our sample of HUNT mothers may not be large enough to effectively partition the genetic variance/covariance in the data between the different possible sources of variation.

**Table 4:** SNP based heritability for each of the traits included in the Genomic SEM models

	Own				Offspring			
**Trait**	**h** ^**2**^	**SE**	**Z**	**p-value**	**h** ^**2**^	**SE**	**Z**	**p-value**
BW	0.089	0.005	19.261	0	0.093	0.005	18.980	< 2 × 10^− 16^
SBP	0.125	0.012	10.767	0	0.030	0.028	1.060	0.145
DBP	0.127	0.012	10.754	0	0.016	0.028	0.587	0.279
Glucose	0.037	0.008	4.357	6.59 × 10^− 6^	0.017	0.030	0.583	0.280
BMI	0.204	0.013	15.669	0	0.073	0.032	2.299	0.011
LDL	0.117	0.015	7.634	1.14 × 10^− 14^	0.044	0.030	1.490	0.068
HDL	0.204	0.032	6.448	5.67 × 10^− 11^	0.017	0.030	0.565	0.286
Triglycerides	0.152	0.024	6.409	7.31 × 10^− 11^	0.086	0.035	2.487	0.006
Total Cholesterol	0.114	0.014	8.246	0	0.057	0.030	1.929	0.027

* Note that these estimates of SNP heritability are likely to be biased because (in particular peri-natal) traits represent a complicated mixture of direct fetal and indirect maternal genetic effects. In addition, maternal (fetal) GWAS need to be corrected (either implicitly or explicitly) for the decrement in power that arises from using maternal (fetal) genotype as a proxy of offspring genotype to estimate direct fetal (indirect maternal) genetic effects- otherwise estimates of SNP heritability will be biased downwards (see de la Fuente et al. 2022 [37] and Supplementary Note 1). Naïve LD score regression analyses do not take into account either of these issues when estimating SNP heritability. In contrast, our model fitted in genomicSEM accounts for both these concerns and should produce asymptotically unbiased estimates of SNP heritability due to both maternal and fetal sources of variation. SNP: Single Nucleotide Polymorphism; BW: Birthweight; SBP: Systolic Blood Pressure; DBP: Diastolic Blood Pressure; Glucose: Non-fasting Glucose; BMI: Body Mass Index; LDL: Low Density Lipoprotein; HDL: High Density Lipoprotein; h^2^: SNP based heritability; SE: Standard Error; Z: Z statistic

Nevertheless, Table 5 presents estimates of the genetic correlation between own/offspring birthweight and own and offspring cardiometabolic traits. There was evidence of a negative genetic correlation between own/offspring birthweight and own blood pressure, and evidence for a positive genetic correlation between own/offspring birthweight and both own and offspring BMI. The large standard errors of the genetic correlations involving the offspring cardiometabolic phenotypes underscore the difficulty in obtaining precise estimates of the (maternally mediated) genetic covariance between birthweight and these later life phenotypes.

**Table 5:** Bivariate LD score regression estimates of the genetic correlation between own birthweight and own and offspring later life cardiometabolic traits

	Own Cardiometabolic Trait				Offspring Cardiometabolic Trait			
**(a) Own BW**								
**Trait**	**r**_**G**_	**SE**	**Z**	**p-value**	**r**_**G**_	**SE**	**Z**	**p-value**
SBP	**-0.138**	**0.039**	**-3.580**	**3.00 × 10** ^**− 4**^	-0.109	0.146	-0.750	0.453
DBP	**-0.120**	**0.035**	**-3.442**	**0.001**	-0.169	0.216	-0.783	0.434
Glucose	-0.103	0.068	-1.513	0.130	0.045	0.186	0.244	0.808
BMI	**0.140**	**0.026**	**5.303**	**1.14 × 10** ^**− 7**^	**0.185**	**0.083**	**2.237**	**0.025**
LDL	0.057	0.051	1.123	0.262	0.068	0.131	0.519	0.604
HDL	-0.001	0.040	-0.025	0.980	-0.359	0.645	-0.557	0.578
Triglycerides	-0.064	0.034	-1.878	0.060	0.032	0.074	0.435	0.664
Total cholesterol	0.027	0.043	0.613	0.540	0.009	0.102	0.093	0.926
**(b) Offspring BW**								
**Trait**	**r**_**G**_	**SE**	**Z**	**p-value**	**r**_**G**_	**SE**	**Z**	**p-value**
SBP	**-0.243**	**0.041**	**-5.916**	**3.30 × 10** ^**− 9**^	-0.416	0.239	-1.738	0.082
DBP	**-0.151**	**0.044**	**-3.463**	**0.001**	-0.341	0.332	-1.026	0.305
Glucose	0.077	0.078	0.987	0.324	0.008	0.168	0.046	0.964
BMI	**0.123**	**0.033**	**3.785**	**2.00 × 10** ^**− 4**^	**0.175**	**0.087**	**2.010**	**0.045**
LDL	0.021	0.051	0.408	0.683	-0.034	0.127	-0.264	0.792
HDL	0.012	0.038	0.307	0.759	-0.597	1.094	-0.546	0.585
Triglycerides	-0.055	0.038	-1.448	0.148	0.023	0.085	0.267	0.789
Total cholesterol	0.016	0.049	0.331	0.741	-0.088	0.104	-0.849	0.396

LD: Linkage Disequilibrium; BW: Birthweight; SBP: Systolic Blood Pressure; DBP: Diastolic Blood Pressure; Glucose: Non-fasting Glucose; BMI: Body Mass Index; LDL: Low Density Lipoprotein; HDL: High Density Lipoprotein; r_G_: genetic correlation; SE: Standard Error; Z: Z statistic

LDL: Low Density Lipoprotein; HDL: High Density Lipoprotein

*95% confidence intervals on the genetic correlations were derived using the delta method. As genetic correlations are computed as functions of genetic covariances and variances, estimates of the sampling variance of the genetic correlation will include additional uncertainty from both genetic covariance and variances

## Genomic SEM Model

In principle, the Genomic SEM model allows us to partition the genetic covariance between traits into offspring and maternally mediated components. However, for most phenotype pairs, this partitioning was uninformative as shown by the wide 95% confidence intervals on the point estimates (Supplementary Tables 3–6). The exceptions were the negative genetic covariance between glucose and birthweight, which appeared to be at least partly mediated through the offspring genome (Table 6), the negative genetic covariance between own systolic blood pressure and offspring birthweight, which was suggestive of a causal effect of maternal systolic blood pressure on offspring birthweight (Supplementary Table 4), and an unexpected negative covariance between the latent genetic factors indexing offspring birthweight and offspring HDL (Table 7). We have also included estimates of genetic correlations in these tables which may be easier to interpret than the raw genetic covariances.

**Table 6:** Estimated genetic covariance between own birthweight and own cardiometabolic traits

	Genetic Covariances			Genetic Correlations*		
	**Effect estimate**	**Lower 95% CI**	**Upper 95% CI**	**Effect estimate**	**Lower 95% CI**	**Upper 95% CI**
**SBP**	-0.005	-0.018	0.009	-0.050	-0.283	0.184
**DBP**	-0.007	-0.019	0.006	-0.071	-0.282	0.140
**Glucose**	**-0.014**	**-0.028**	**-0.001**	-0.238	-0.608	0.131
**BMI**	0.013	-0.001	0.027	0.119	-0.059	0.298
**HDL**	0.001	-0.013	0.016	0.013	-0.178	0.205
**LDL**	0.002	-0.010	0.015	0.031	-0.251	0.314
**Triglycerides**	-0.009	-0.021	0.004	-0.099	-0.334	0.137
**Total cholesterol**	-0.002	-0.014	0.011	-0.024	-0.317	0.268

CI: Confidence interval; SBP: Systolic Blood Pressure; DBP: Diastolic Blood Pressure; Glucose: Non-fasting Glucose; BMI: Body Mass Index;

**Table 7:** Estimated genetic covariance between offspring birthweight and offspring cardiometabolic traits

	Genetic Covariances			Genetic Correlations*		
	**Effect estimate**	**Lower 95% CI**	**Upper 95% CI**	**Effect estimate**	**Lower 95% CI**	**Upper 95% CI**
Offspring SBP	-0.012	-0.032	0.007	-0.883	-1^#^	1^#^
Offspring DBP	-0.005	-0.027	0.016	-0.547	-1^#^	1^#^
Offspring Glucose	-0.007	-0.028	0.014	-0.132	-0.716	0.451
Offspring BMI	0.006	-0.017	0.029	0.184	-0.972	1^#^
Offspring HDL	**-0.024**	**-0.044**	**-0.003**	-0.726	-1^#^	1^#^
Offspring LDL	-0.006	-0.027	0.014	-0.276	-1^#^	1^#^
Offspring Triglycerides	0.002	-0.020	0.025	0.035	-0.396	0.465
Offspring Total cholesterol	-0.013	-0.033	0.007	-0.390	-1^#^	1^#^

CI: Confidence interval; SBP: Systolic Blood Pressure; DBP: Diastolic Blood Pressure; Glucose: Non-fasting Glucose; BMI: Body Mass Index; LDL: Low Density Lipoprotein; HDL High Density Lipoprotein

*95% confidence intervals on the genetic correlations were derived using the delta method. As genetic correlations are computed as functions of genetic covariances and variances, estimates of the sampling variance of the genetic correlation will include additional uncertainty from both genetic covariance and variances. ^#^The estimated confidence interval was out of bounds

## Discussion

In this manuscript we introduce a new statistical model that is capable in theory of partitioning the genetic covariation between traits into maternal and offspring contributions. Our model builds upon previous work by our group and others that has demonstrated how the genetic *variance* in a trait can be informatively decomposed into maternal and offspring components [38–40]. These previous approaches used structural equation modeling of individual level genome-wide genotype data from mother-offspring pairs [39, 41]) or parent-child trios [40] to estimate maternal and offspring genetic variance components. In contrast, the present method uses genomic SEM [15] applied to summary results GWAS data to decompose genetic variation and covariation between traits into maternal and offspring components. It has an advantage over previous approaches in that it neither requires complete mother-offspring pairs, nor individual level genotype data. In addition, any cryptic relatedness that may exist between different mother-offspring dyads is automatically taken into account by LD score regression estimates of the total genetic variance and covariance. In contrast, how best to handle inter-pair relatedness can present a thorny problem for individual level G-REML approaches like M-GCTA and trio-GCTA in that such pairs can bias estimates of SNP heritability / variance components, whilst their removal can often result in substantial decrements to sample size and statistical power [38, 42].

We applied our genomic SEM to summary results GWAS data from the HUNT Study in order to partition the genetic covariance between birthweight and later life cardiometabolic outcomes into maternal and offspring mediated components. Our motivation was that partitioning might be informative with respect to the genesis of the well-known correlation between birthweight and cardiometabolic disease [1, 4, 7, 43]. Furthermore, the creation and application of genetic approaches like the one espoused in this manuscript could provide a useful complement to traditional observational epidemiological approaches in investigating the validity of the Barker and the Developmental Origin of Health and Disease Hypotheses. For example, the existence of a significant (negative) maternally mediated genetic covariance between birthweight and cardiometabolic phenotypes would strongly imply the existence of intrauterine mechanisms consistent with the Barker Hypothesis.

Overall, although the LD score regression analyses produced evidence for a significant genetic correlation between own/offspring birthweight and several later life traits in the HUNT Study (e.g. blood pressure, BMI), in most cases our genomic SEM did not have the statistical power to informatively resolve most covariances into maternal and offspring components (despite the large sample size of HUNT). This was likely due to the high negative correlation between competing maternal and fetal parameter estimates that are typical of these sorts of models [44], as well as the low magnitude of the observed genetic covariance between the variables (although in theory significant maternal and offspring components can still be resolved if they act in opposite directions [14]). Previous epidemiological studies have reported statistically significant albeit low magnitude correlations between birth weight and future cardiometabolic traits (e.g. |r| < 0.15) [45, 46]. The phenotypic correlations reported in the HUNT cohort were even lower than these. Part of the reason for the low observed covariances may be because the present study includes many young individuals in the GWAS of cardiometabolic variables. It may be that cardiometabolic changes develop over a long period time, with only minor variation in early adulthood and cumulatively more distinct patterns in later life. For example, in the UK Biobank study, genetic correlations between birthweight and cardiometabolic risk factors (where individuals are older on average than HUNT) appear to be much larger than in the present study (|r| > 0.2) [9, 11]. In this regard, there is an ongoing challenge of obtaining cohorts that include large numbers of maternal genotypes and offspring phenotypes (i.e. which are necessary to partition effects into maternal and offspring components) where the offspring are old enough to have developed cardiometabolic disease [41]. Statistical approaches where maternal/parental genotypes are imputed from (elderly) relative pairs provide a potential way to assuage this problem [47, 48].

It is noteworthy that the authors of LD score regression suggest that only traits that have strong evidence of SNP heritability (i.e. Z scores > 4) are likely to yield reliable estimates of genetic correlations [36]. Whilst birthweight (i.e. own birthweight and offspring birthweight) and one’s own cardiometabolic phenotypes all exhibited strong evidence of SNP heritability, this was not the case for offspring cardiometabolic traits. Table 4 shows that the estimated SNP heritability for the offspring cardiometabolic phenotypes was low (and the Z scores < 4), despite the expectation that these GWAS results should contain a signal for own genotype (i.e. since maternal genotype and offspring genotype are correlated 0.5) regardless of whether there exists any genuine maternal genetic effects on offspring phenotype. The lack of strong genetic signals here suggests that our GWAS of offspring cardiometabolic traits may be underpowered and that we should be circumspect with respect to any conclusions drawn from our study.

These caveats aside, we did observe some weak evidence for a negative maternally mediated genetic covariance between offspring birthweight and offspring HDL, between SBP and offspring birthweight, and between glucose and own birthweight. A significant (negative) genetic covariance between SBP and offspring birthweight is consistent with a causal relationship between maternal SBP and offspring birthweight, which has been reported using Mendelian randomization in several studies [11, 49]. Likewise, genetic pleiotropy through an individual’s own genome could feasibly explain the negative genetic correlation between glucose and own birthweight (i.e. alleles which predispose to higher circulating glucose also predispose to poorer glucose utilization and lower birthweight), and is consistent with the results of genetic correlations and Mendelian randomization studies in the UK Biobank and Fetal Insulin Hypothesis models of birthweight more generally [14]. The negative maternally mediated genetic covariance between offspring birthweight and offspring HDL is a novel observation suggestive of Barker Hypothesis type mechanisms, but the p-value is marginal and requires replication in other cohorts.

Our genomic SEM involves a number of simplifications. First, we do not include a latent genetic variable for paternal effects. In theory, it would be possible to add latent variables indexing paternal genetics [50] and the relevant paternal GWAS to the genomic SEM, however, we chose not to do this because (a) there is little evidence that paternal genetic effects contribute meaningfully to variation in birthweight or cardiometabolic disease, and (b) the inclusion of paternal terms would have further decreased power to detect variance components involving offspring mediated effects. That being said, modelling father-offspring pairs separately (in a similar genomic SEM) could provide a useful control comparison where intrauterine mechanisms are suspected as being important. Second our model does not allow for the influence of assortative mating. (Positive) assortment exerts a myriad of complicated effects on the genome including increasing the genetic variance and inducing correlations between trait relevant loci across the genome [51]. However, there is little evidence to suggest that phenotypic assortment is an important component influencing variation in birthweight or cardiometabolic traits. Recent work by Keller and colleagues have illustrated [52, 53] how assortment can be incorporated into a structural equation modeling framework involving individual level data and polygenic risk scores. How to model assortment and its effects on offspring phenotypes in a genomic SEM framework is an active area of research for our and other groups. Finally, we note that whilst in principle our model could be extended to simultaneously investigate maternal and offspring GWAS of more than two variables, we have chosen not to do so here, as the number of parameters and their interpretation quickly becomes cumbersome/complicated.

## Conclusion

In conclusion, we have developed a new method using genomic SEM that can decompose genetic variances and covariances into maternal and offspring components using summary results data from GWAS of mothers and their offspring. Application of this model to investigate the relationship between birthweight and later life cardiometabolic phenotypes in the HUNT study mostly yielded inconclusive findings due to lack of statistical power. However, we did find some evidence for maternally mediated effects of systolic blood pressure on offspring birthweight, and pleiotropy between birthweight and non-fasting glucose mediated through the offspring genome which is consistent with previous investigations. Our results underscore the genetic links between birthweight and cardiometabolic phenotypes in later life.

## Electronic Supplementary Material

Below is the link to the electronic supplementary material.

Supplementary Material 1

Supplementary Material 2

Supplementary Material 3

Supplementary Material 4

## Data Availability

(Data Transparency) The empirical datasets used with the HUNT study will be archived with the study and will be made available to individuals who obtain the necessary permissions from the study’s Data Access Committee. Due to privacy issues, access to individual-level data requires permission from the HUNT Study, the Medical Birth Registry of Norway and the regional committee for medical research ethics. Requirements for access to data from the HUNT Study are described at www.ntnu.edu/hunt.

## Code Availability

Example code is provided in Supplementary Note 2.
