# Supplementary material for: Using Genomic Structural Equation Modeling to Partition the Genetic Covariance Between Birthweight and Cardiometabolic Risk Factors into Maternal and Offspring Components in the Norwegian HUNT Study
Source: Behav Genet. 2022 Nov 2;53(1):40–52. doi: 10.1007/s10519-022-10116-9 (PMC9823066; doi:10.1007/s10519-022-10116-9)
Supplement: Supplementary file 1 — Supplementary Material 1 [file 10519_2022_10116_MOESM1_ESM.docx]

Supplementary Note 1

Recall that that variance explained by a single additive biallelic SNP is:

$$\sigma_{q}^{2}=2p(1-p)\beta^{2}$$

where *p* is that increaser allele frequency and *β* the effect on the phenotype of each increaser allele.

If we use maternal genotype as a proxy for offspring genotype, the population regression coefficient will be halved, which implies that the variance explained by the locus will be one quarter the original size i.e.

$$\sigma_{q}^{2}=2p(1-p)(\frac{1}{2}{\beta)}^{2}=\frac{1}{2}p(1-p)\beta^{2}$$

Under the alternative hypothesis of true genetic association, the non-centrality parameter (NCP) for the test of association of the regression of phenotype on genotype (i.e. number of copies of the allele) is given by:

$${\mathrm{NCP}=(\frac{\beta}{SE(\beta)})}^{2}=\frac{{2p(1-p)N\beta}^{2}}{(\sigma_{p}^{2}-2p(1-p)\beta^{2})}$$

where $\sigma_{p}^{2}$ is the trait variance and *N* the sample size.

If *β* decreases by a multiple of 0.5 (i.e. by using maternal genotype as a proxy for own genotype), the sample size needs to increase approximately fourfold to obtain the same non-centrality parameter and hence power to detect association.

We note that similar conclusions were reached by de la Fuente et al (2022)(1), who also point out that maternal (fetal) GWAS need to be corrected (either implicitly or explicitly) for the decrement in power that arises from using maternal (fetal) genotype as a proxy of offspring genotype to estimate direct fetal (indirect maternal) genetic effects- otherwise estimates of SNP heritability produced by LD score regression may be biased downwards.

Similar to the model described in de la Fuente et al. (2022)(1), our method doesn’t require manual correction of GWAS summary results statistics before input into genomicSEM/LD score regression (indeed, similar to de la Fuente et al, it requires uncorrected summary GWAS results statistics in order to obtain asymptotically unbiased parameter estimates). However, we note that our model differs from de la Fuente et al. in a number of important ways including that we estimate the variance of latent maternal and fetal genetic factors (de la Fuente et al. only estimate one common latent genetic factor representing direct genetic effects), and we fix the values of the path coefficients between the latent and observed variables to one or 0.5 (i.e. whereas de la Fuente et al. estimate these path coefficients). Our fixing of these paths is justified in that we are trying to estimate the genetic variance (and covariance) explained by maternal and fetal genetic effects, and our model requires us to constrain these paths to these values in order to informatively partition genetic variance into maternal and fetal sources of variation. In contrast, the focus in de la Fuentes et al. is to estimate the direct effect of SNPs (and associated SNP heritability), and maternal and paternal GWAS are only included in the analysis because their inclusion provides extra statistical power to estimate these effects (i.e. the authors do not examine indirect genetic effects).

1. de la Fuente J, Grotzinger AD, Marioni RE, Nivard MG, Tucker-Drob EM. Integrated analysis of direct and proxy genome wide association studies highlights polygenicity of Alzheimer’s disease outside of the APOE region. PLOS Genetics. 2022;18(6):e1010208.
