# Supplementary material for: Using Genomic Structural Equation Modeling to Partition the Genetic Covariance Between Birthweight and Cardiometabolic Risk Factors into Maternal and Offspring Components in the Norwegian HUNT Study
Source: Behav Genet. 2022 Nov 2;53(1):40–52. doi: 10.1007/s10519-022-10116-9 (PMC9823066; doi:10.1007/s10519-022-10116-9)
Supplement: Supplementary file 2 — Supplementary Material 2 [file 10519_2022_10116_MOESM2_ESM.docx]

**Supplementary Note 2 – Example code**

library(devtools)

library(GenomicSEM)

files <- c("OwnBirthweight.txt", "Maternal-offspringBirthweight.txt",

"OwnLaterLifeTrait.txt", "Mother_OffspringLaterLifeTrait.txt") **#Path to summary statistics**

hm3 <- "w_hm3.noMHC.snplist" #**SNP reference list**

trait.names <- c("OwnBW","Maternal1offBW","OwnLaterLifeTrait","MaternalLaterLifeTrait") **#Trait names**

N <- c() **#Fill inn N individuals in each summary statistics**

munge(files=files,hm3=hm3, trait.names=trait.names, N=N) **#Munging summary statistics specified above**

traits <- c("OwnBirthweight.sumstats.gz", " Maternal-offspringBirthweight.sumstats.gz",
 "OwnLaterLifeTrait.sumstats.gz", "MaternalLaterLifeTrait.sumstats.gz") **#Munged summary statistics**

sample.prev <- c(NA,NA,NA,NA) **#Setting prevalence to NA due to continuous trait**

population.prev <- c(NA,NA,NA,NA) **#Setting prevalence to NA due to continuous trait**

ld <- “eur_w_ld_chr/" **#Path to LD score reference panel**

wld <- "eur_w_ld_chr/" **#Path to LD score reference panel**

LD_result <- ldsc(traits=traits, sample.prev=sample.prev, population.prev=population.prev, ld=ld, wld=wld,trait.names=trait.names) **#Run LD score**

save(LD_result, file=" LD_result.RData") **#Save result**

**## Run genomic SEM model**

load("LD_result.RData")

covstruc <- LD_result

**## Specifying Model: (using SBP as later life trait example phenotype):**

**# Trait names specified above must match the names given to the summary statistics specified in model**

BW_SBP_model <- ' **#Model name**

BW =~ .5*Maternal1offBW **#Loading 0.5 of maternal birthweight summary statistics on BW latent variable**

BW =~ 1*OwnBW **#Loading own birthweight summary statistics on BW latent variable**

OffspringBW =~ 1*Maternal1offBW **#Loading maternal birthweight summary statistics on OffspringBW latent variable**

OffspringBW =~ .5*OwnBW **#Loading 0.5 of own birthweight summary statistics on OffspringBW latent variable**

SBP =~ 1*OwnSBP **#Loading own SBP summary statistics on SBP latent variable**

SBP =~ .5*MaternalSBP **#Loading 0.5 maternal SBP summary statistics on SBP latent variable**

OffspringSBP =~ .5*OwnSBP **#Loading 0.5 of own SBP summary statistics on OffspringSBP latent variable**

OffspringSBP =~ 1*MaternalSBP **#Loading maternal SBP summary statistics on OffspringSBP latent variable**

Maternal1offBW ~~ 0*Maternal1offBW + 0*OwnBW + 0*OwnSBP + 0*MaternalSBP **#Setting all the residual covariance’s to zero**

OwnBW ~~ 0*OwnBW + 0*OwnSBP + 0*MaternalSBP

OwnSBP ~~ 0*OwnSBP + 0*MaternalSBP

MaternalSBP ~~ 0*MaternalSBP

BW ~~ cov12*OffspringBW **#Estimating covariances between latent variables**

BW ~~ cov13*SBP

BW ~~ cov14*OffspringSBP

OffspringBW ~~ cov23*SBP

OffspringBW ~~ cov24*OffspringSBP

SBP ~~ cov34*OffspringSBP

BW ~~var1*BW **#Estimating variances of the latent variables**

OffspringBW ~~ var2*OffspringBW

SBP ~~ var3*SBP

OffspringSBP ~~ var4*OffspringSBP

corBW_OffspringBW := cov12/(sqrt(var1)*sqrt(var2)) **#Calculating correlations between the latent variables**

corBW_SBP := cov13/(sqrt(var1)*sqrt(var3))

corBW_OffspringSBP := cov14/(sqrt(var1)*sqrt(var4))

corSBP_OffspringBW := cov23/(sqrt(var2)*sqrt(var3))

corOffspringSBP_OffspringBW := cov24/(sqrt(var2)*sqrt(var4))

corSBP_OffspringSBP := cov34/(sqrt(var3)*sqrt(var4))

'

Estimation <- "DWLS" **#Specifying the estimation method to use**

std.lv <- FALSE **#Specifying whether variances of latent variables should be set to 1**

BW_SNP_results <- usermodel(covstruc=covstruc, model=BW_SBP_model,estimation=estimation,std.lv=std.lv, CFIcalc = TRUE) **#Running model**

BW_SNP_results **#Printing results of model run**
