# Supplementary material for: Using Genomic Structural Equation Modeling to Partition the Genetic Covariance Between Birthweight and Cardiometabolic Risk Factors into Maternal and Offspring Components in the Norwegian HUNT Study
Source: Behav Genet. 2022 Nov 2;53(1):40–52. doi: 10.1007/s10519-022-10116-9 (PMC9823066; doi:10.1007/s10519-022-10116-9)
Supplement: Supplementary file 3 — Supplementary Material 3 [file 10519_2022_10116_MOESM3_ESM.docx]

**Supplementary Note 3 – Manhattan and QQ plots**

**
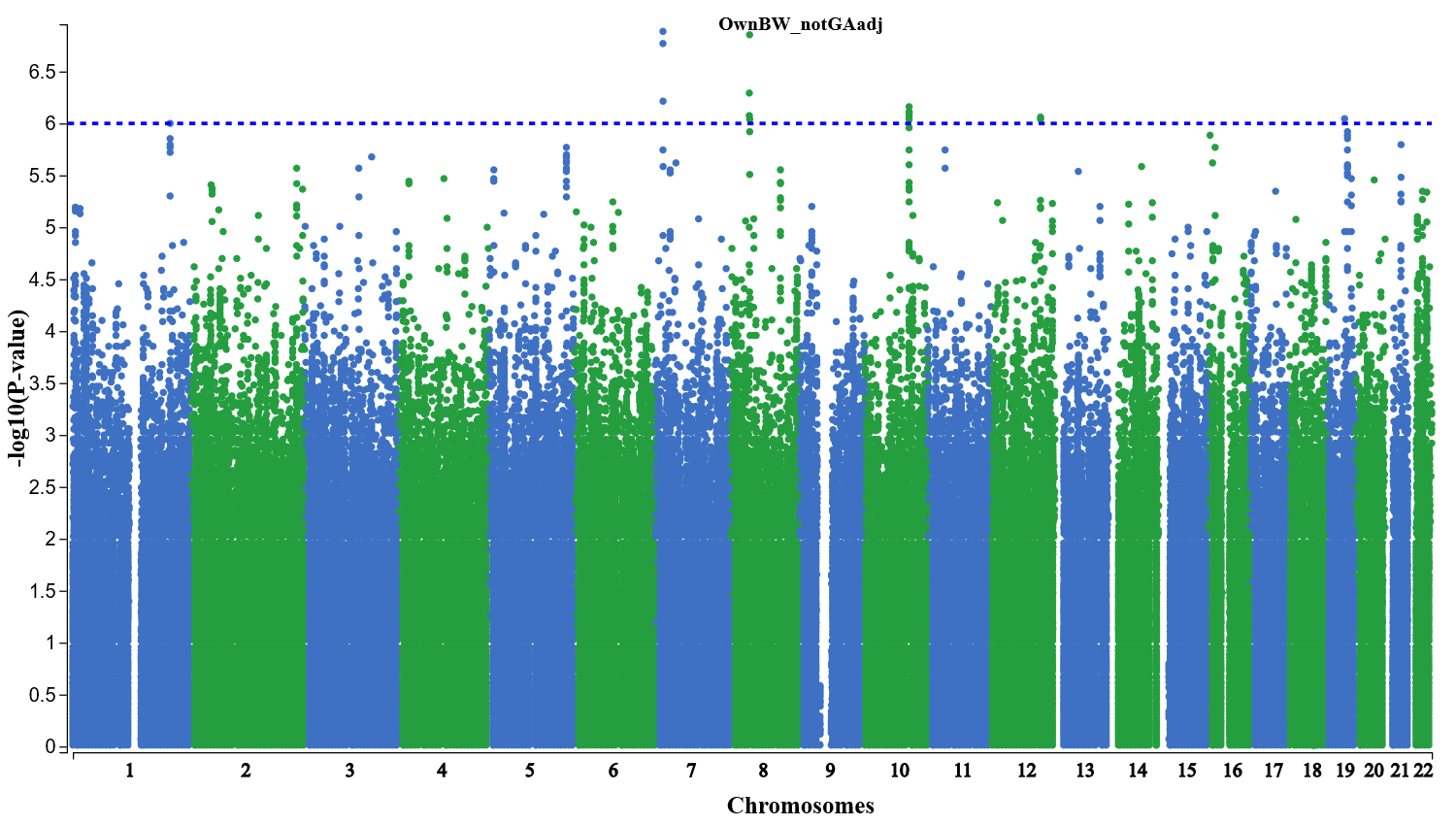
**

**Supplementary Figure 1: Manhattan plot of own birthweight in the HUNT Study.** Each dot represents a genetic variant. The X-axis shows the position of the genetic variants on the chromosomes and the Y-axis displays the –log10 p-value. The red and blue dashed lines represent genome-wide (p = 5 x 10^-8^) and suggestive levels of significance (p = 1 x 10^-6^).

**
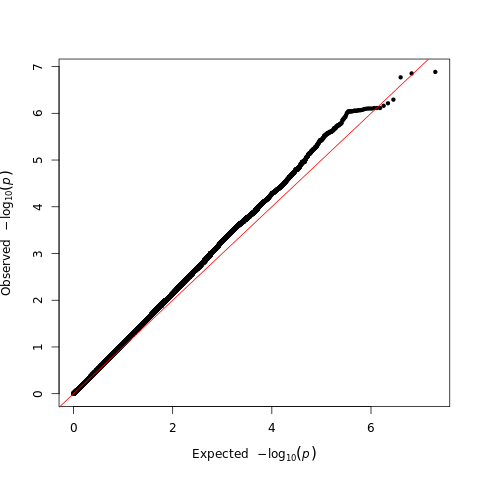
**

**Supplementary Figure 2: QQ plot of own birthweight.**

**
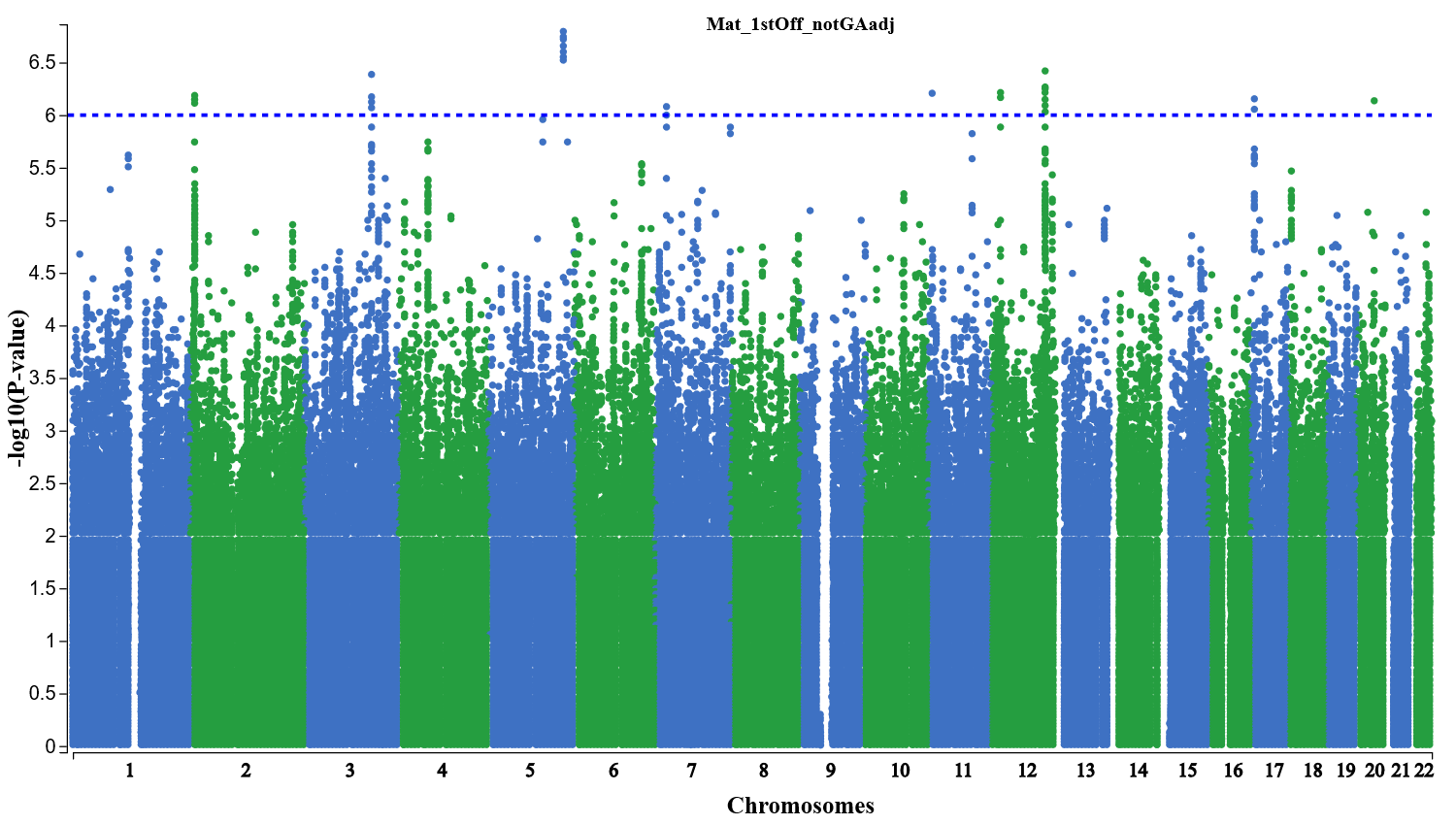
**

**Supplementary Figure 3: Manhattan plot of offspring birthweight in the HUNT Study.** Each dot represents a genetic variant. The X-axis shows the position of the genetic variants on the chromosomes and the Y-axis displays the –log10 p-value. The red and blue dashed lines represent genome-wide (p = 5 x 10^-8^) and suggestive levels of significance (p = 1 x 10^-6^).


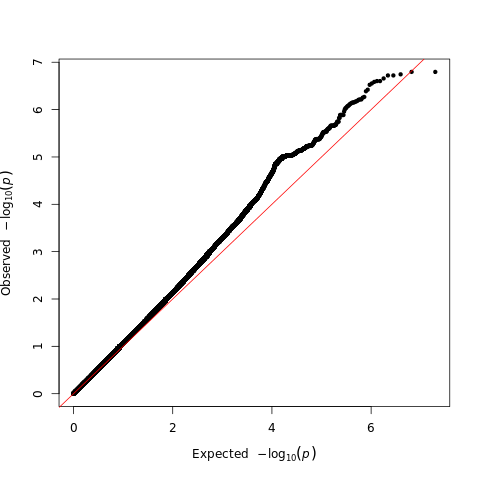


**Supplementary Figure 4: QQ plot of offspring birthweight.**


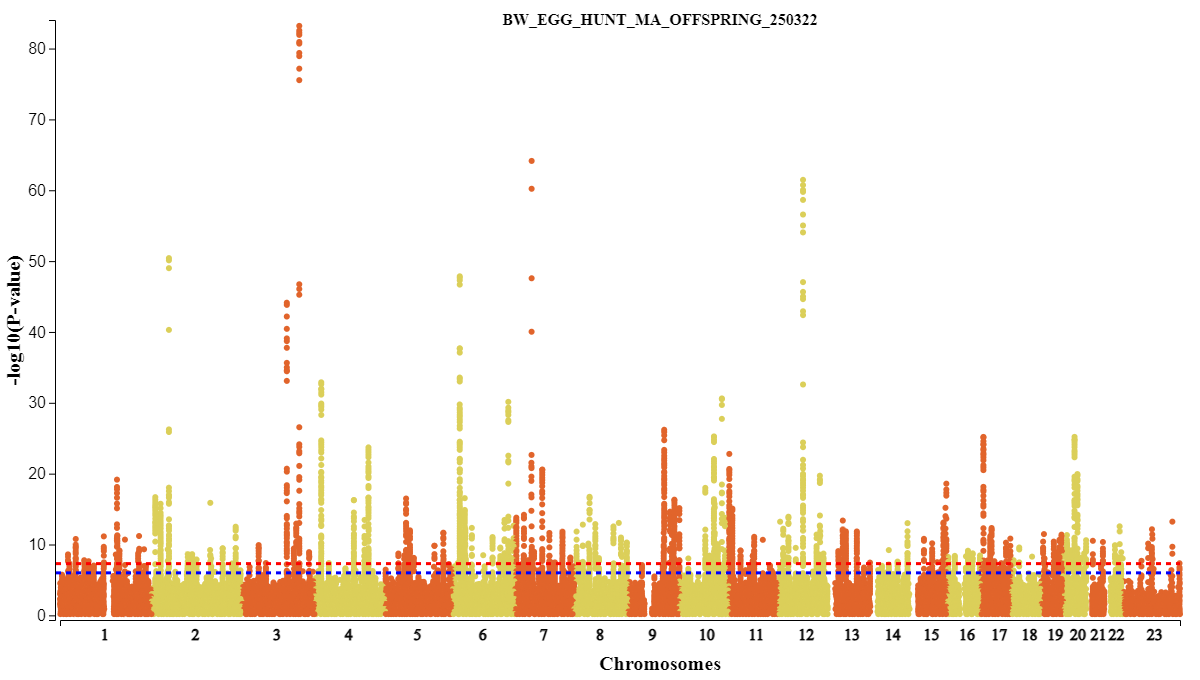
**Supplementary Figure 5: Manhattan plot of own birthweight in the HUNT Study and EGG meta-analysis.** Each dot represents a genetic variant. The X-axis shows the position of the genetic variants on the chromosomes and the Y-axis displays the –log10 p-value. The red and blue dashed lines represent genome-wide (p = 5 x 10^-8^) and suggestive levels of significance (p = 1 x 10^-6^).


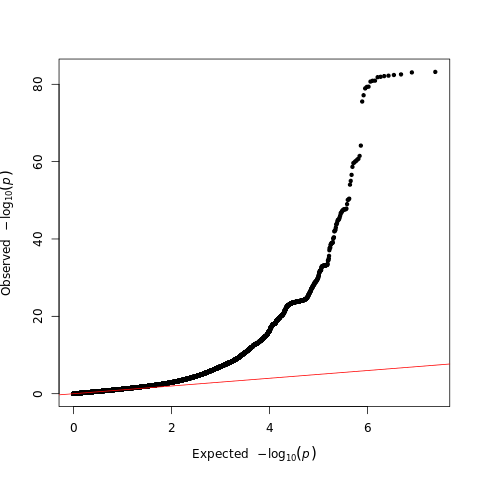


**Supplementary Figure 6: QQ plot of meta-analysis own birthweight.**


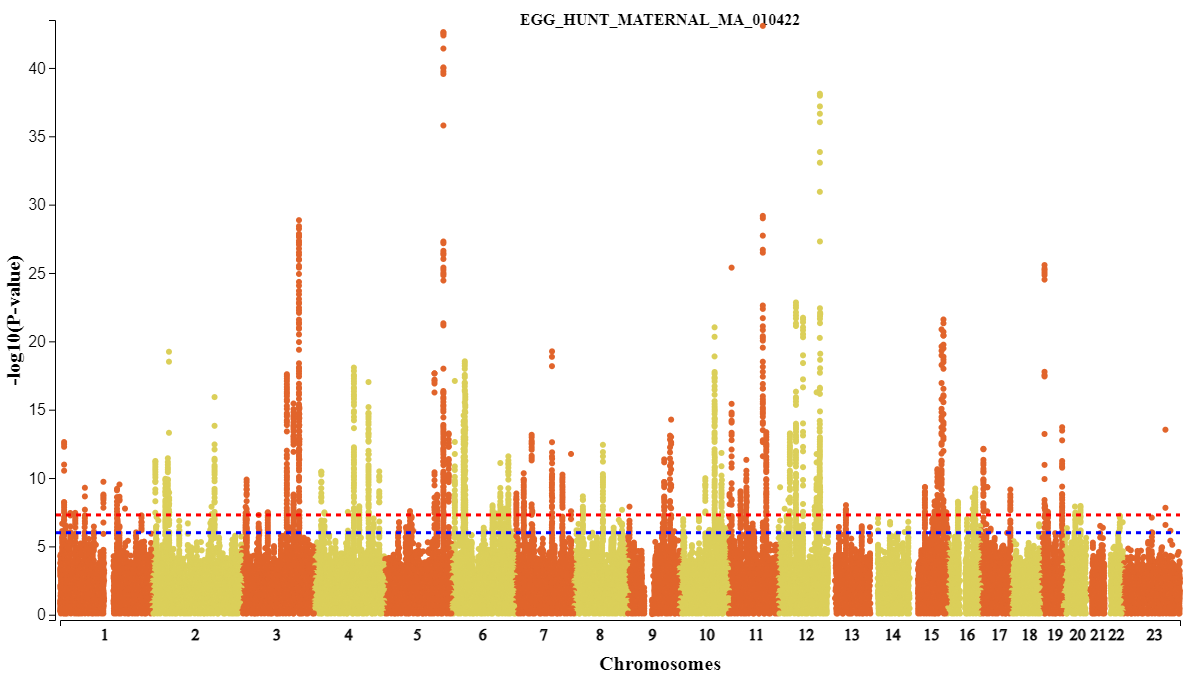
**Supplementary Figure 7: Manhattan plot of offspring birthweight in the HUNT Study and EGG meta-analysis.** Each dot represents a genetic variant. The X-axis shows the position of the genetic variants on the chromosomes and the Y-axis displays the –log10 p-value. The red and blue dashed lines represent genome-wide (p = 5 x 10^-8^) and suggestive levels of significance (p = 1 x 10^-6^).


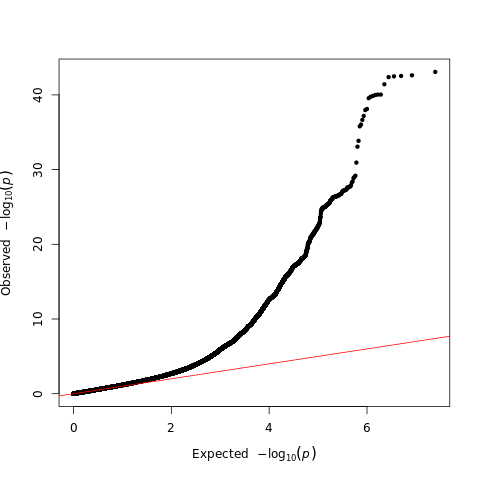


**Supplementary Figure 8: QQ plot of meta-analysis offspring birthweight.**

**
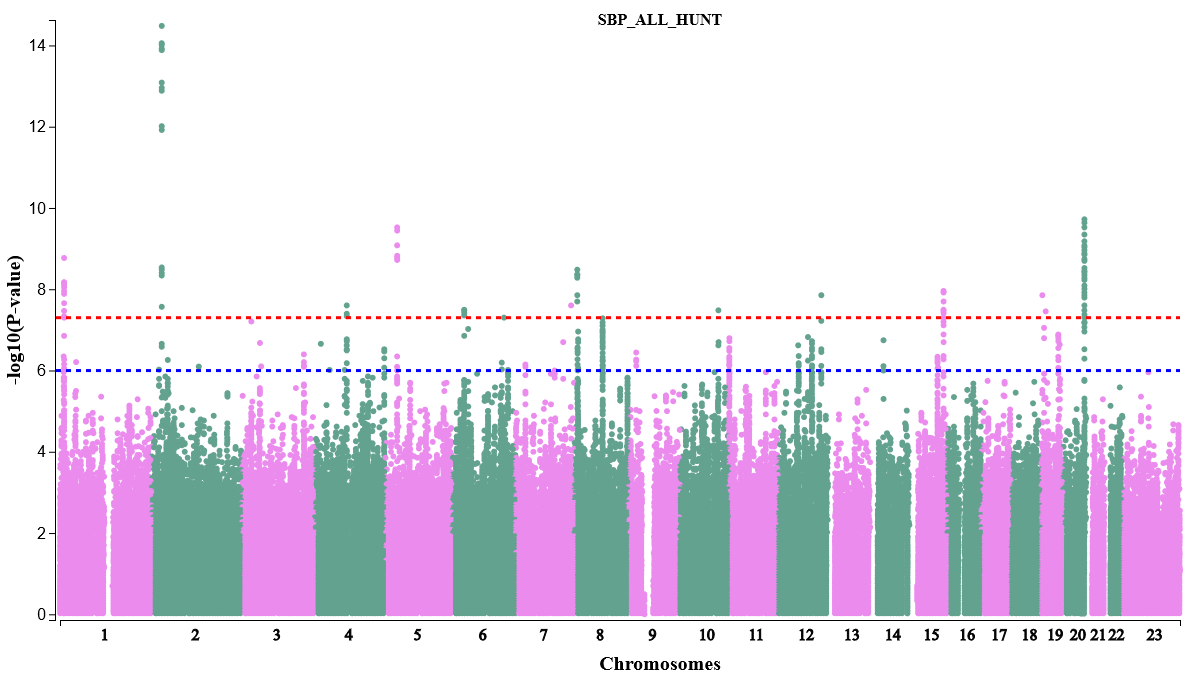
**

**Supplementary Figure 9: Manhattan plot of own systolic blood pressure in the HUNT Study.** Each dot represents a genetic variant. The X-axis shows the position of the genetic variants on the chromosomes and the Y-axis displays the –log10 p-value. The red and blue dashed lines represent genome-wide (p = 5 x 10^-8^) and suggestive levels of significance (p = 1 x 10^-6^).


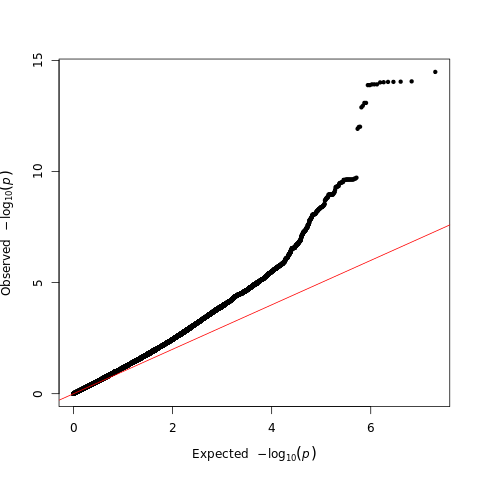


**Supplementary Figure 10: QQ plot of own systolic blood pressure.**

**
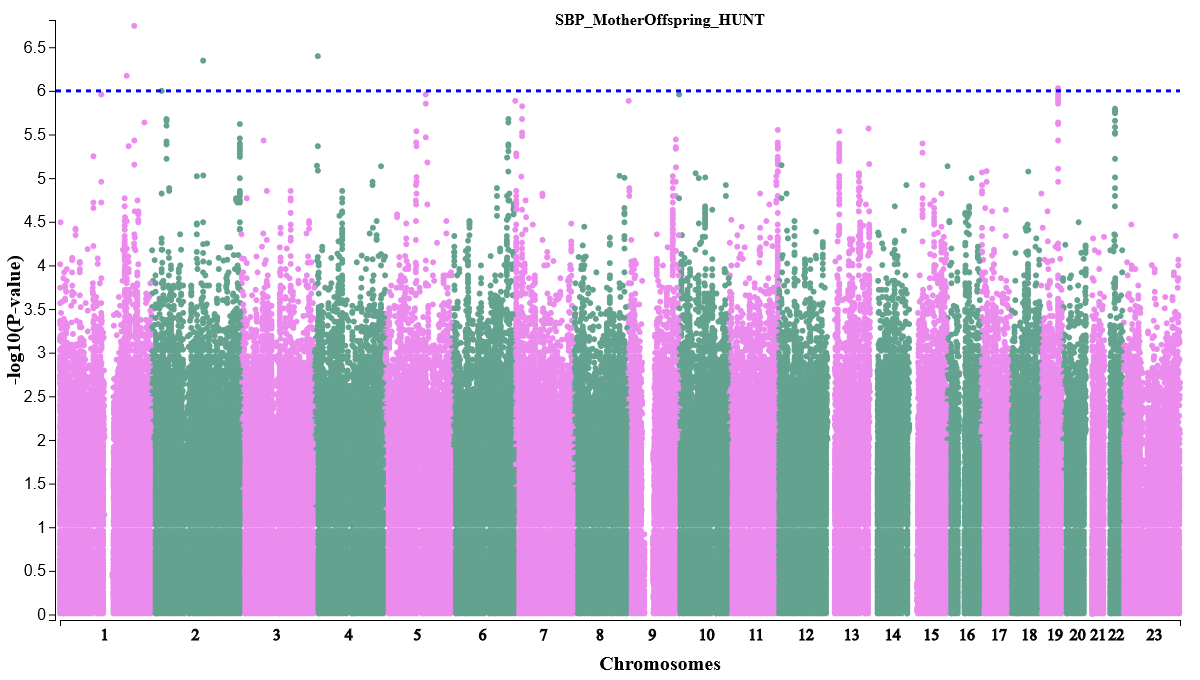
**

**Supplementary Figure 11: Manhattan plot of offspring systolic blood pressure in the HUNT Study.** Each dot represents a genetic variant. The X-axis shows the position of the genetic variants on the chromosomes and the Y-axis displays the –log10 p-value. The red and blue dashed lines represent genome-wide (p = 5 x 10^-8^) and suggestive levels of significance (p = 1 x 10^-6^).


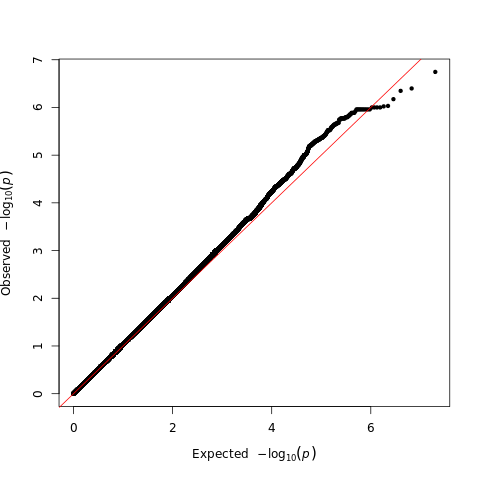


**Supplementary Figure 12: QQ plot of offspring systolic blood pressure.**

**
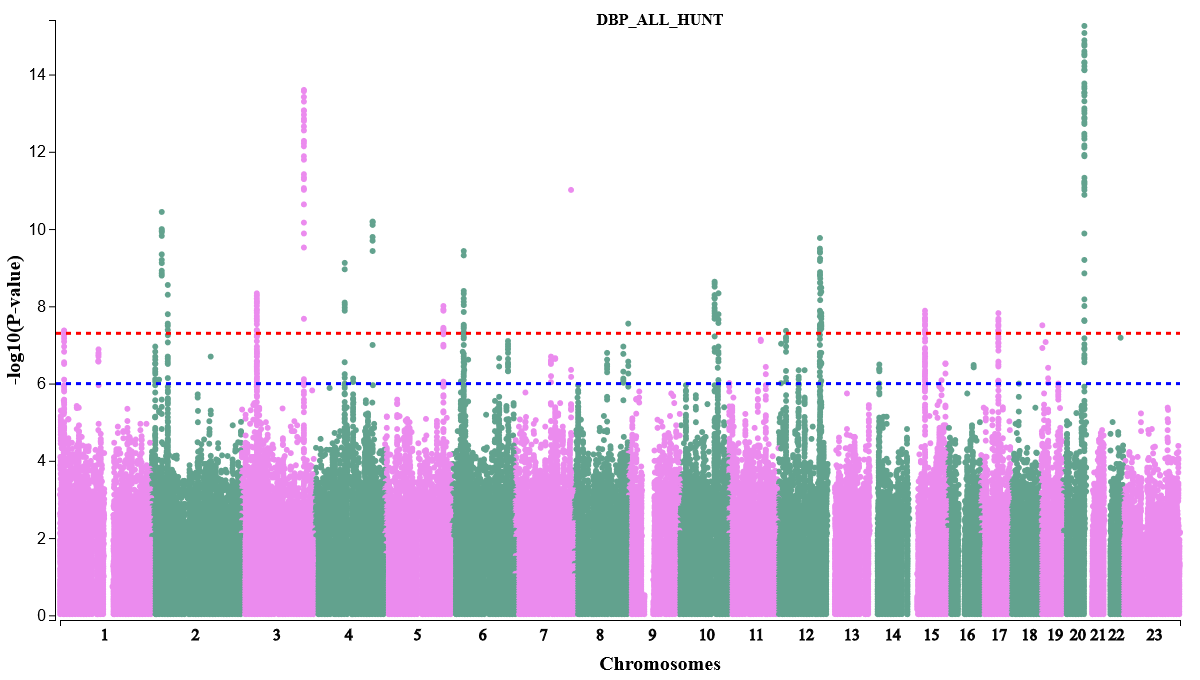
**

**Supplementary Figure 13: Manhattan plot of own diastolic blood pressure in the HUNT Study.** Each dot represents a genetic variant. The X-axis shows the position of the genetic variants on the chromosomes and the Y-axis displays the –log10 p-value. The red and blue dashed lines represent genome-wide (p = 5 x 10^-8^) and suggestive levels of significance (p = 1 x 10^-6^).


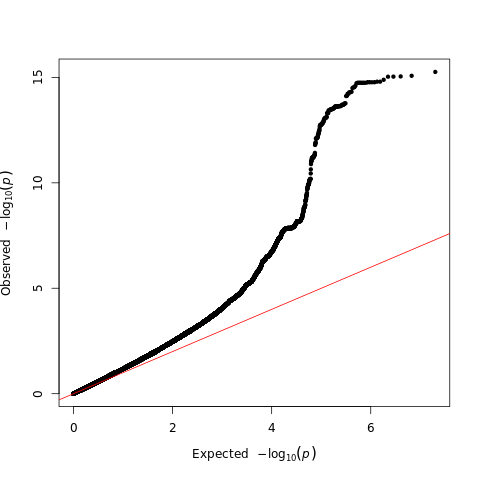


**Supplementary Figure 14: QQ plot of own diastolic blood pressure.**

**
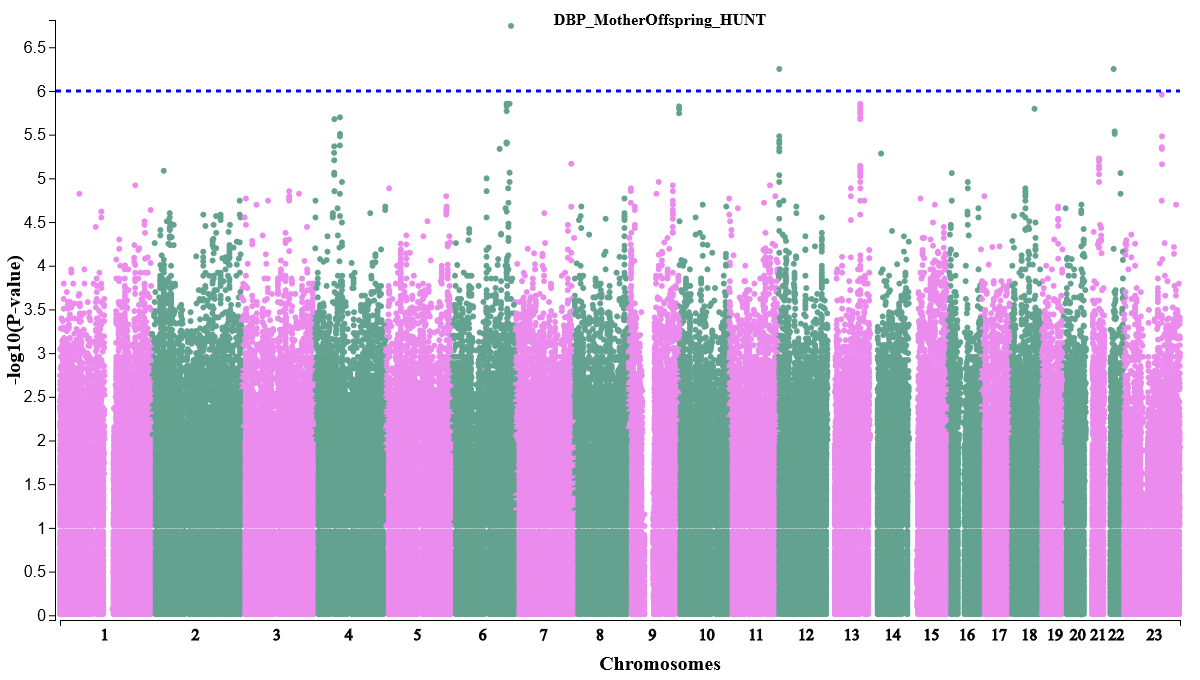
**

**Supplementary Figure 15: Manhattan plot of offspring diastolic blood pressure in the HUNT Study.** Each dot represents a genetic variant. The X-axis shows the position of the genetic variants on the chromosomes and the Y-axis displays the –log10 p-value. The red and blue dashed lines represent genome-wide (p = 5 x 10^-8^) and suggestive levels of significance (p = 1 x 10^-6^).


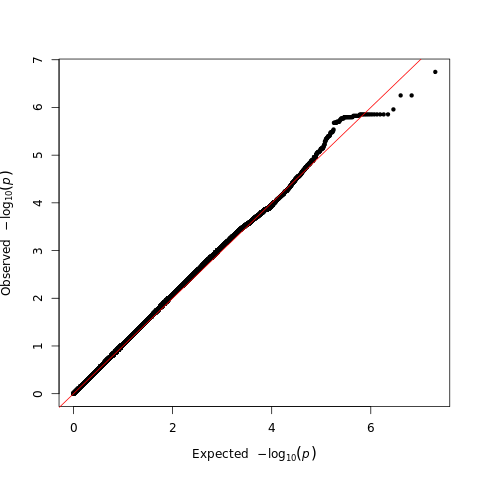


**Supplementary Figure 16: QQ plot of offspring diastolic blood pressure.**

**
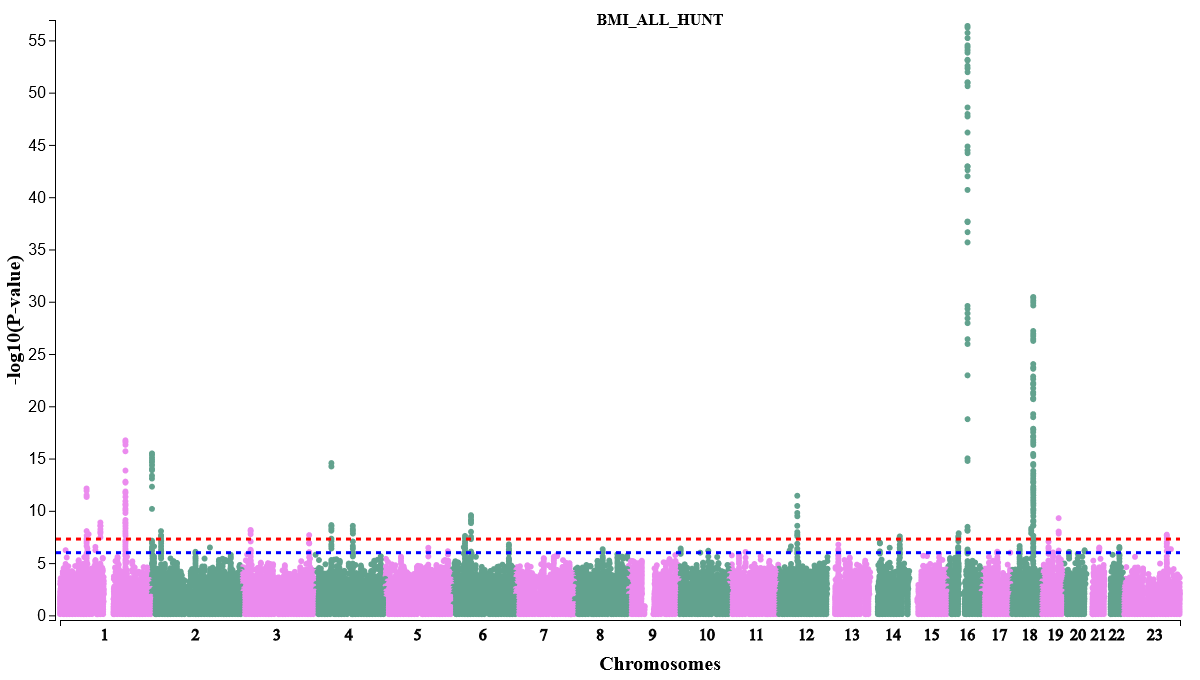
**

**Supplementary Figure 17: Manhattan plot of own body mass index in the HUNT Study.** Each dot represents a genetic variant. The X-axis shows the position of the genetic variants on the chromosomes and the Y-axis displays the –log10 p-value. The red and blue dashed lines represent genome-wide (p = 5 x 10^-8^) and suggestive levels of significance (p = 1 x 10^-6^).


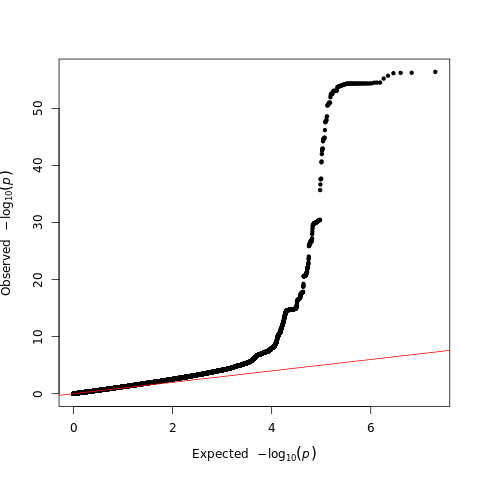


**Supplementary Figure 18: QQ plot of own body mass index.**

**
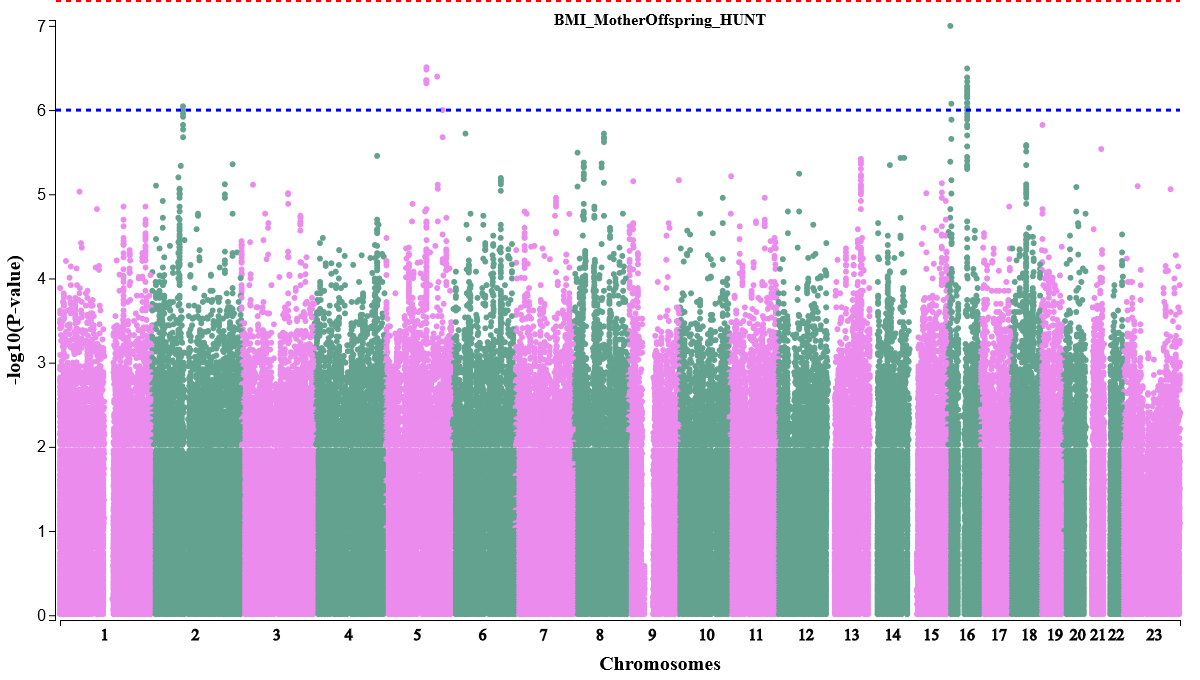
**

**Supplementary Figure 19: Manhattan plot of offspring body mass index in the HUNT Study.** Each dot represents a genetic variant. The X-axis shows the position of the genetic variants on the chromosomes and the Y-axis displays the –log10 p-value. The red and blue dashed lines represent genome-wide (p = 5 x 10^-8^) and suggestive levels of significance (p = 1 x 10^-6^).


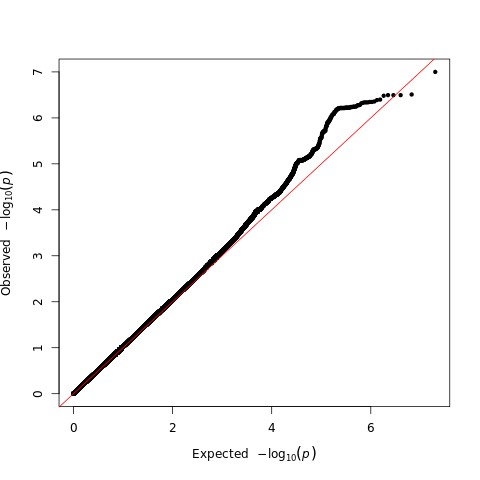


**Supplementary Figure 20: QQ plot of offspring body mass index.**

**
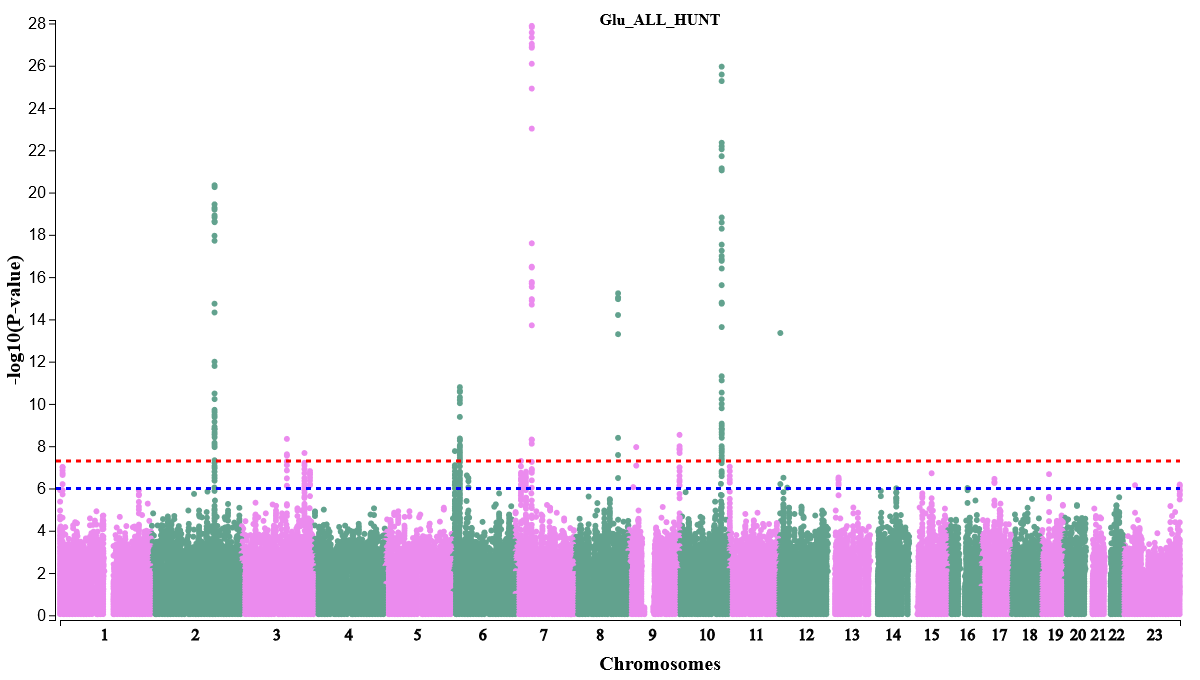
**

**Supplementary Figure 21: Manhattan plot of own non-fasting glucose in the HUNT Study.** Each dot represents a genetic variant. The X-axis shows the position of the genetic variants on the chromosomes and the Y-axis displays the –log10 p-value. The red and blue dashed lines represent genome-wide (p = 5 x 10^-8^) and suggestive levels of significance (p = 1 x 10^-6^).


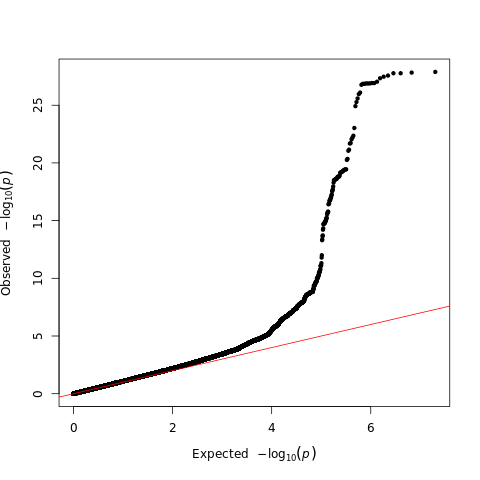


**Supplementary Figure 22: QQ plot of own non-fasting glucose.**

**
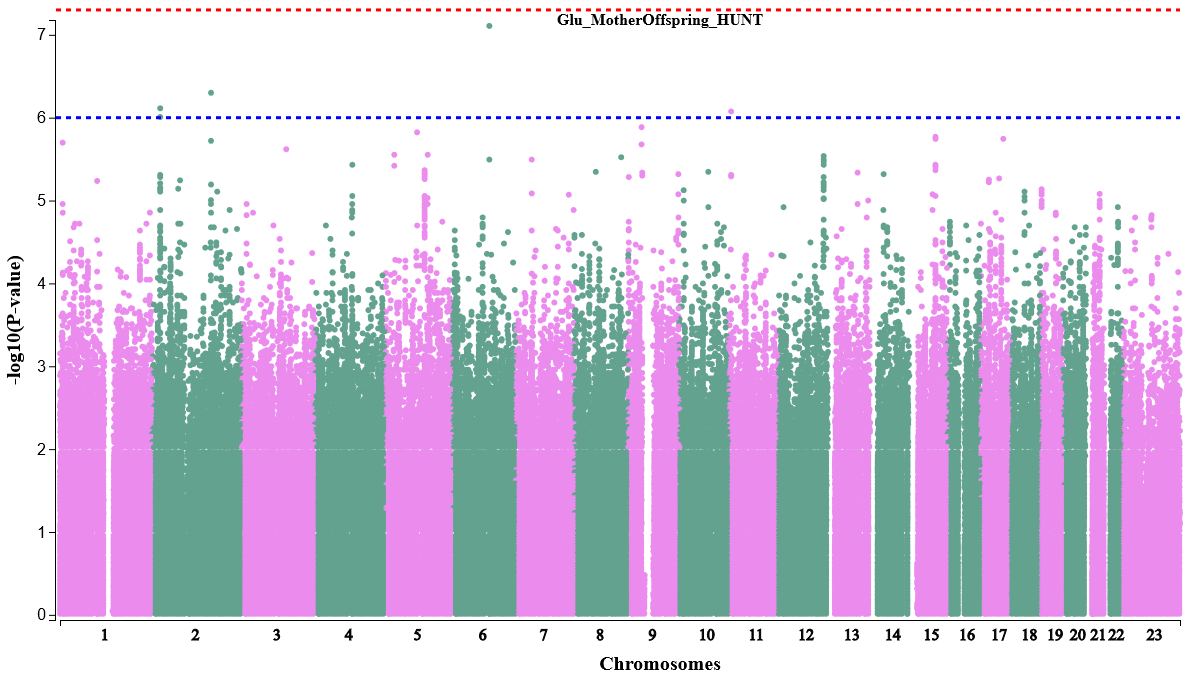
**

**Supplementary Figure 23: Manhattan plot of offspring non-fasting glucose in the HUNT Study.** Each dot represents a genetic variant. The X-axis shows the position of the genetic variants on the chromosomes and the Y-axis displays the –log10 p-value. The red and blue dashed lines represent genome-wide (p = 5 x 10^-8^) and suggestive levels of significance (p = 1 x 10^-6^).


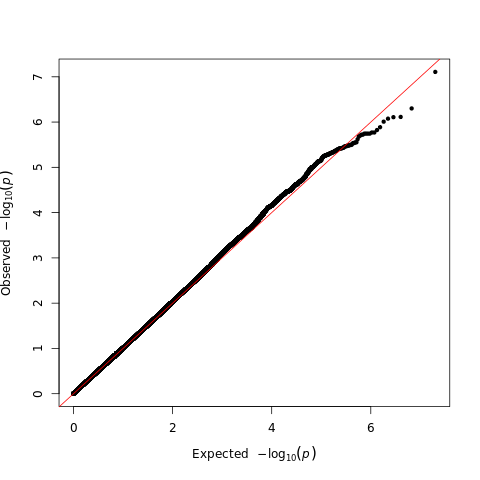


**Supplementary Figure 24: QQ plot of offspring non-fasting glucose.**

**
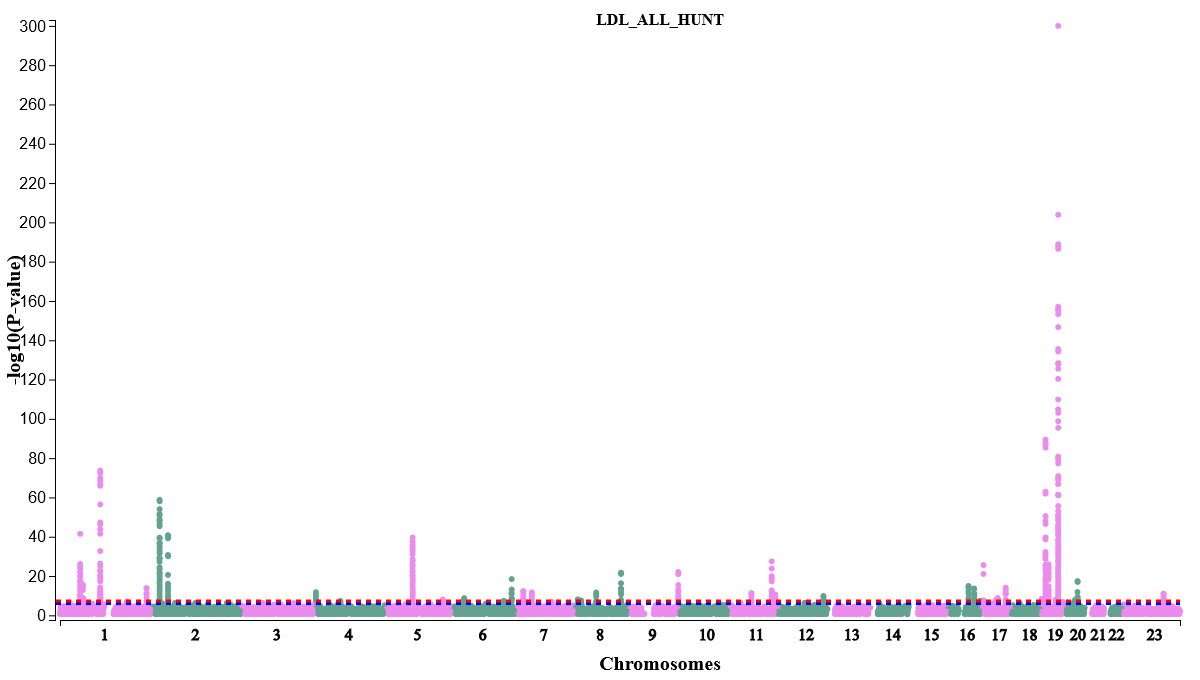
**

**Supplementary Figure 25: Manhattan plot of own Low Density Lipoprotein in the HUNT Study.** Each dot represents a genetic variant. The X-axis shows the position of the genetic variants on the chromosomes and the Y-axis displays the –log10 p-value. The red and blue dashed lines represent genome-wide (p = 5 x 10^-8^) and suggestive levels of significance (p = 1 x 10^-6^).


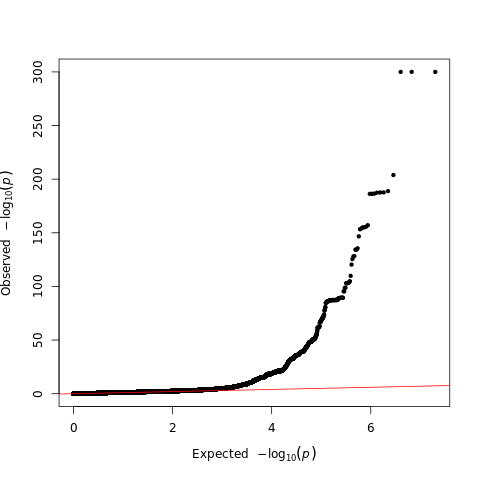


**Supplementary Figure 26: QQ plot of own Low Density Lipoprotein.**

**
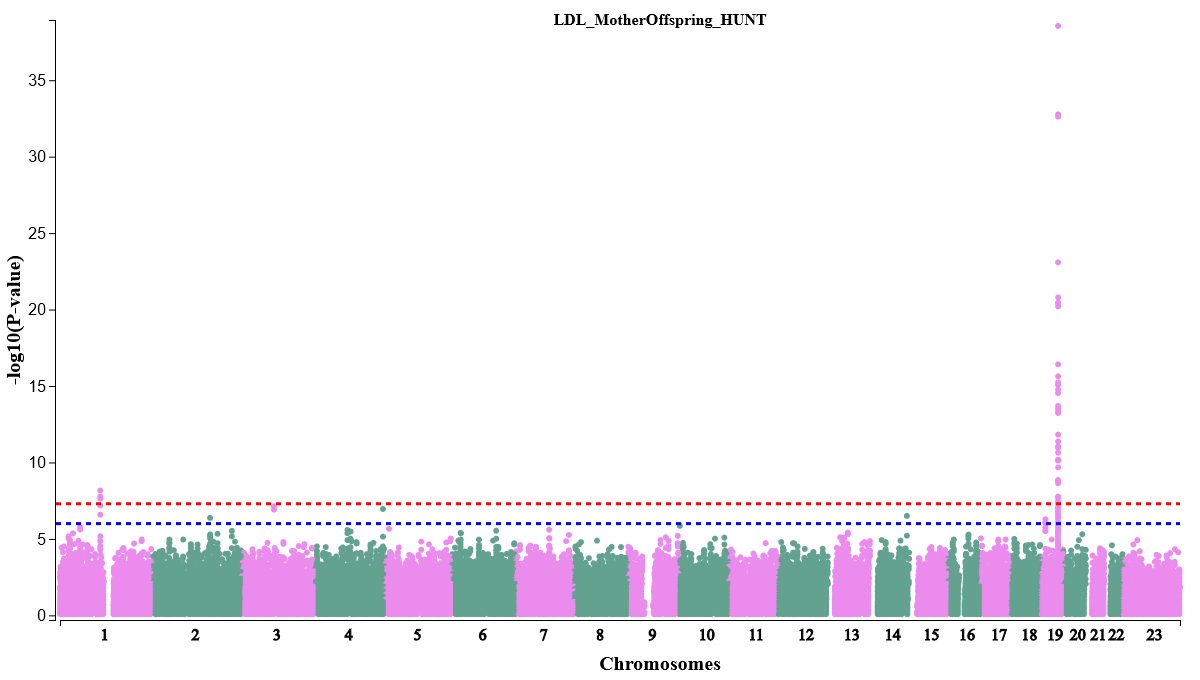
**

**Supplementary Figure 27: Manhattan plot of offspring Low Density Lipoprotein in the HUNT Study.** Each dot represents a genetic variant. The X-axis shows the position of the genetic variants on the chromosomes and the Y-axis displays the –log10 p-value. The red and blue dashed lines represent genome-wide (p = 5 x 10^-8^) and suggestive levels of significance (p = 1 x 10^-6^).
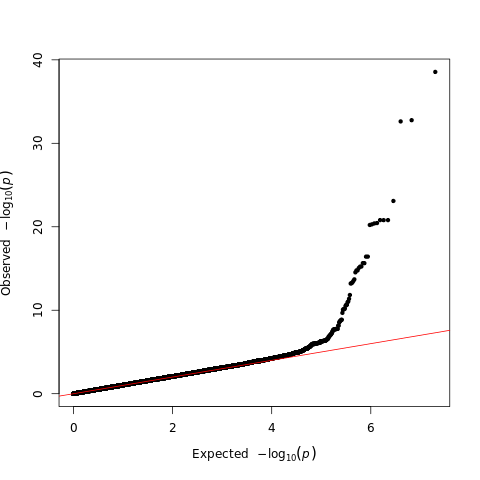


**Supplementary Figure 28: QQ plot of offspring Low Density Lipoprotein.**

**
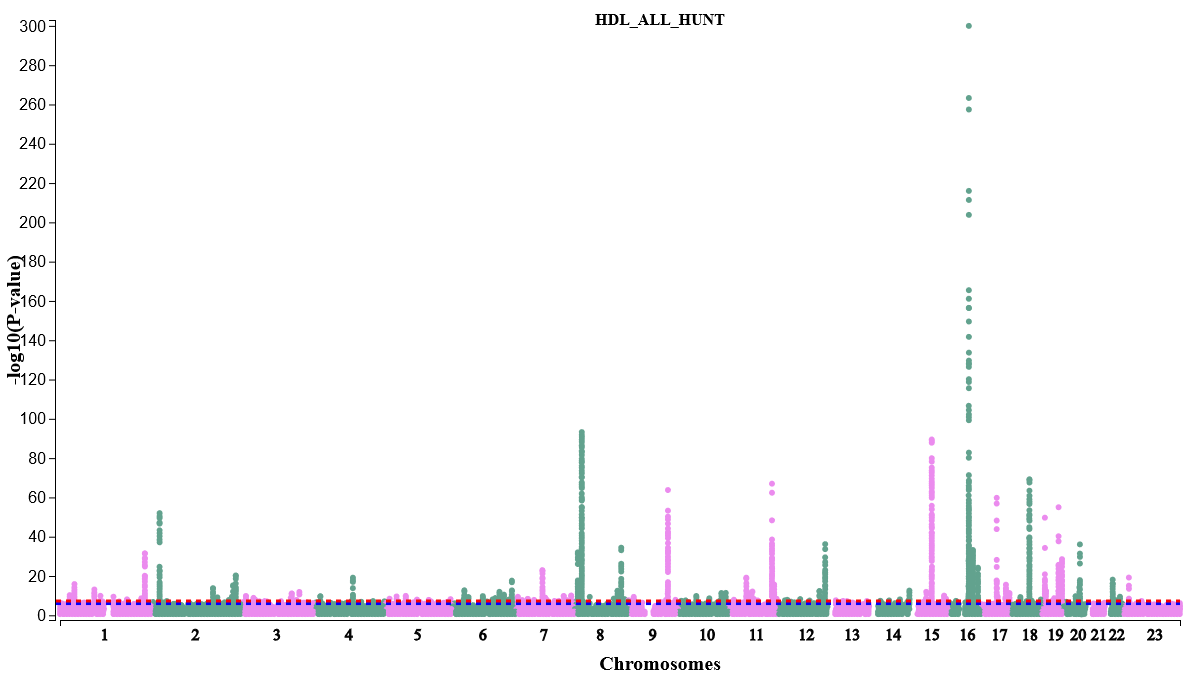
**

**Supplementary Figure 29: Manhattan plot of own High Density Lipoprotein in the HUNT Study.** Each dot represents a genetic variant. The X-axis shows the position of the genetic variants on the chromosomes and the Y-axis displays the –log10 p-value. The red and blue dashed lines represent genome-wide (p = 5 x 10^-8^) and suggestive levels of significance (p = 1 x 10^-6^).


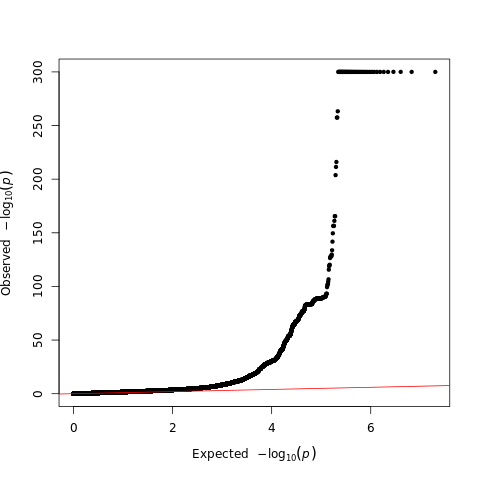


**Supplementary Figure 30: QQ plot of own High Density Lipoprotein.**

**
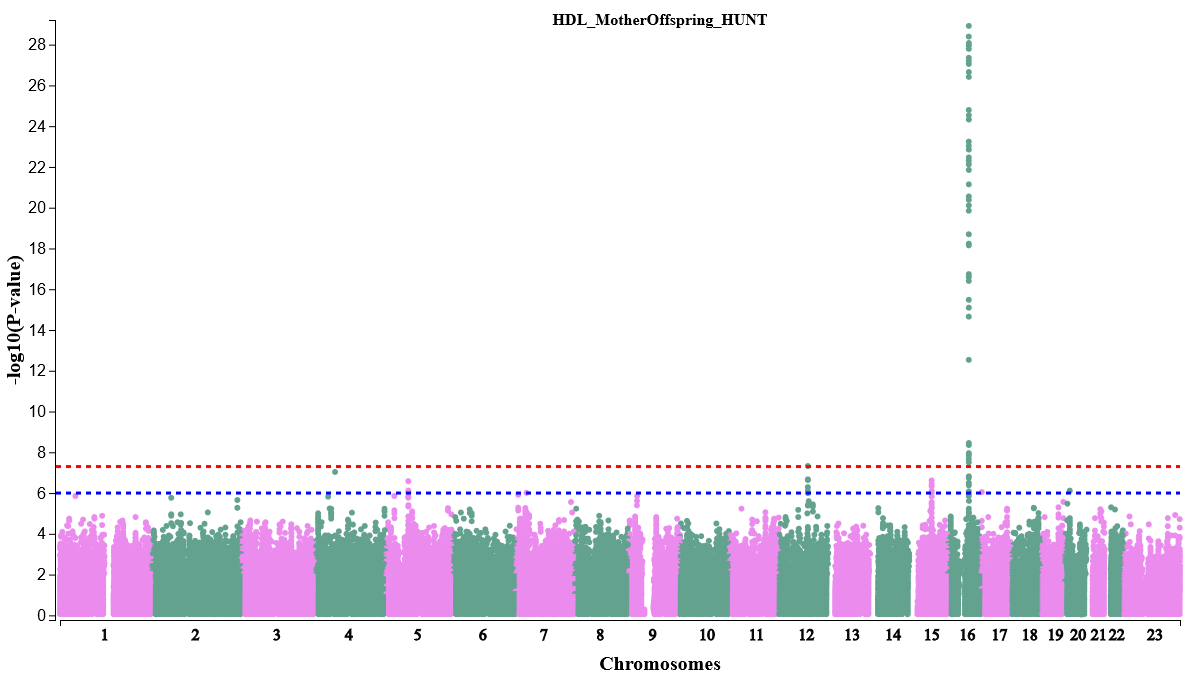
**

**Supplementary Figure 31: Manhattan plot of offspring High Density Lipoprotein in the HUNT Study.** Each dot represents a genetic variant. The X-axis shows the position of the genetic variants on the chromosomes and the Y-axis displays the –log10 p-value. The red and blue dashed lines represent genome-wide (p = 5 x 10^-8^) and suggestive levels of significance (p = 1 x 10^-6^).


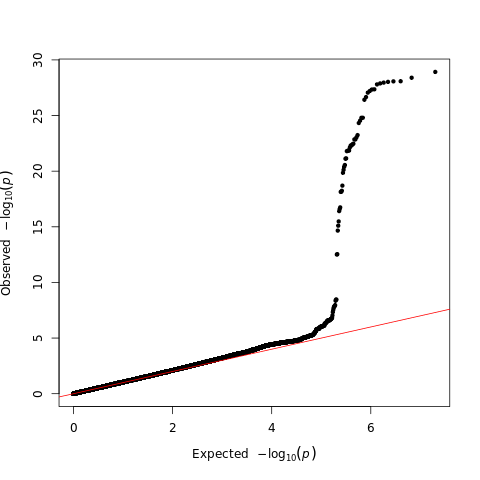


**Supplementary Figure 32: QQ plot of offspring High Density Lipoprotein.**

**
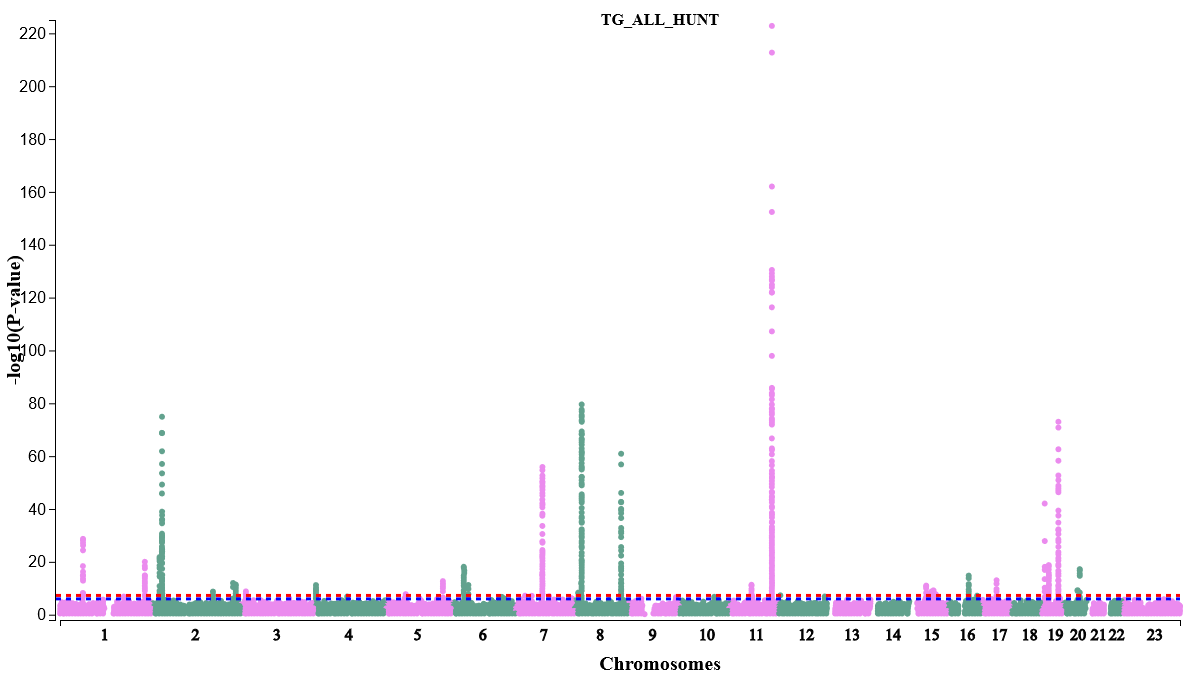
**

**Supplementary Figure 33: Manhattan plot of own Triglycerides in the HUNT Study.** Each dot represents a genetic variant. The X-axis shows the position of the genetic variants on the chromosomes and the Y-axis displays the –log10 p-value. The red and blue dashed lines represent genome-wide (p = 5 x 10^-8^) and suggestive levels of significance (p = 1 x 10^-6^).


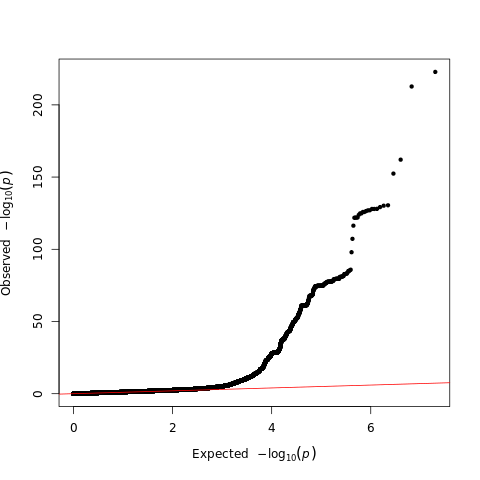


**Supplementary Figure 34: QQ plot of own Triglycerides.**

**
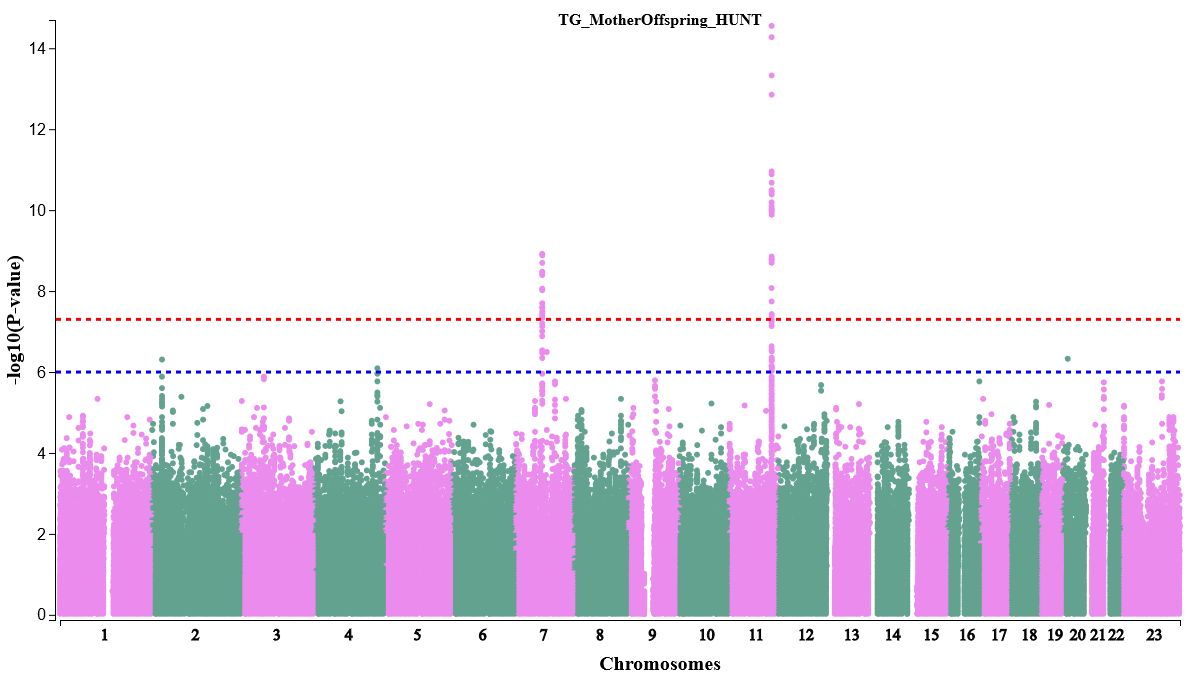
**

**Supplementary Figure 35: Manhattan plot of offspring Triglycerides in the HUNT Study.** Each dot represents a genetic variant. The X-axis shows the position of the genetic variants on the chromosomes and the Y-axis displays the –log10 p-value. The red and blue dashed lines represent genome-wide (p = 5 x 10^-8^) and suggestive levels of significance (p = 1 x 10^-6^).


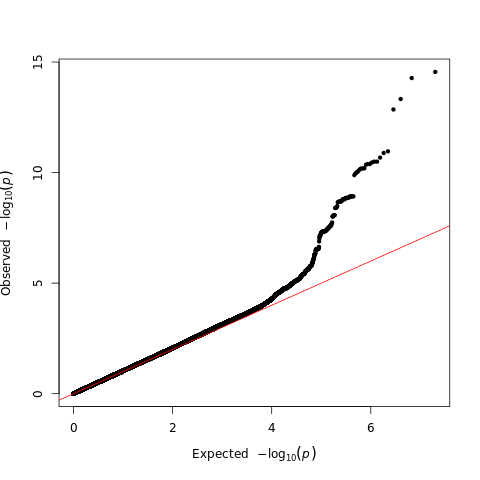


**Supplementary Figure 36: QQ plot of offspring Triglycerides.**

**
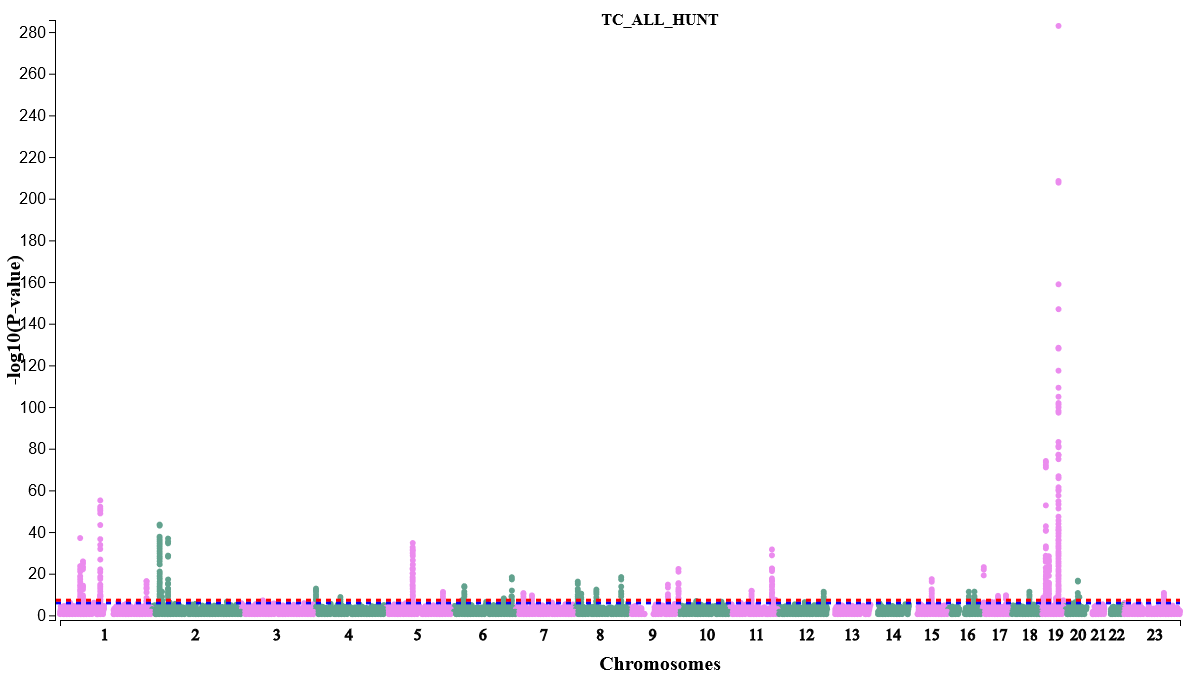
**

**Supplementary Figure 37: Manhattan plot of own Total Cholesterol in the HUNT Study.** Each dot represents a genetic variant. The X-axis shows the position of the genetic variants on the chromosomes and the Y-axis displays the –log10 p-value. The red and blue dashed lines represent genome-wide (p = 5 x 10^-8^) and suggestive levels of significance (p = 1 x 10^-6^).


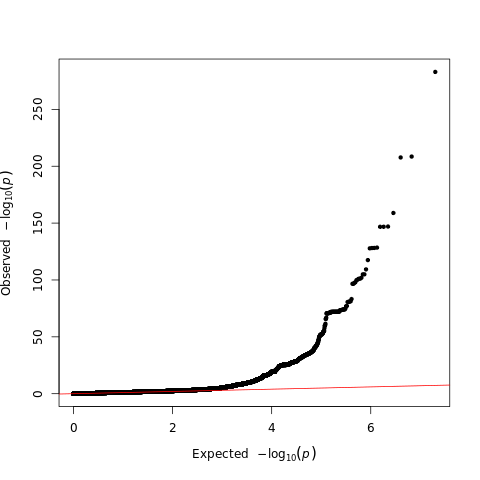


**Supplementary Figure 38: QQ plot of own Total Cholesterol.**

**
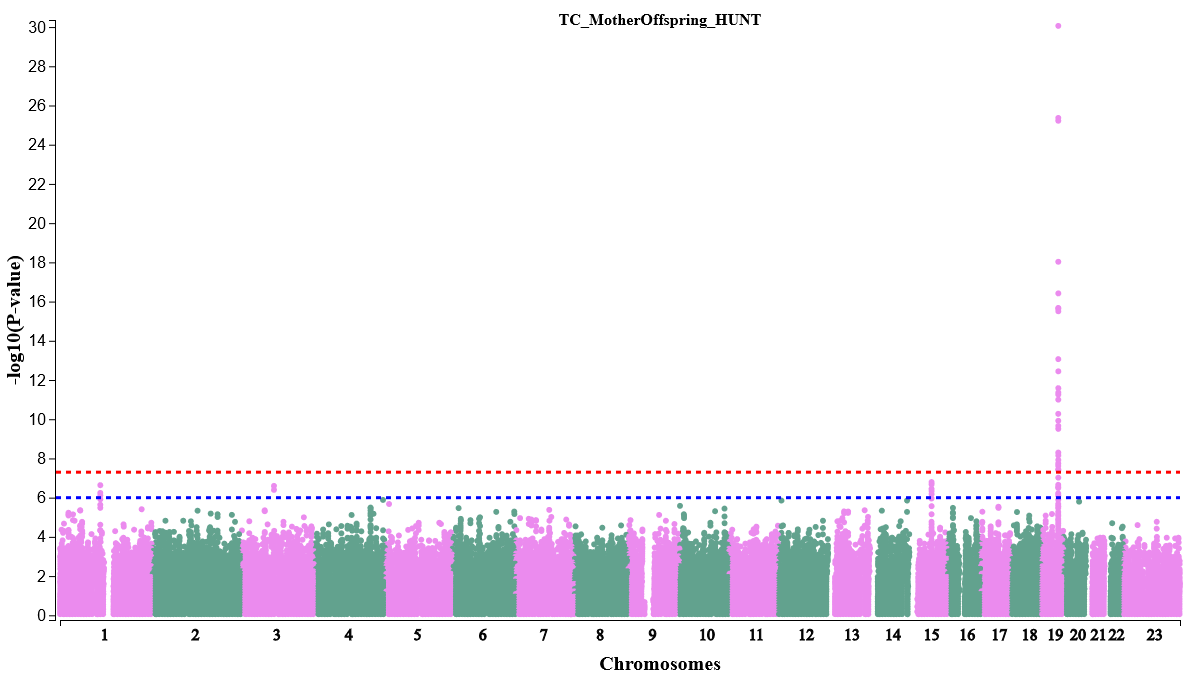
**

**Supplementary Figure 39: Manhattan plot of offspring Total Cholesterol in the HUNT Study.** Each dot represents a genetic variant. The X-axis shows the position of the genetic variants on the chromosomes and the Y-axis displays the –log10 p-value. The red and blue dashed lines represent genome-wide (p = 5 x 10^-8^) and suggestive levels of significance (p = 1 x 10^-6^).


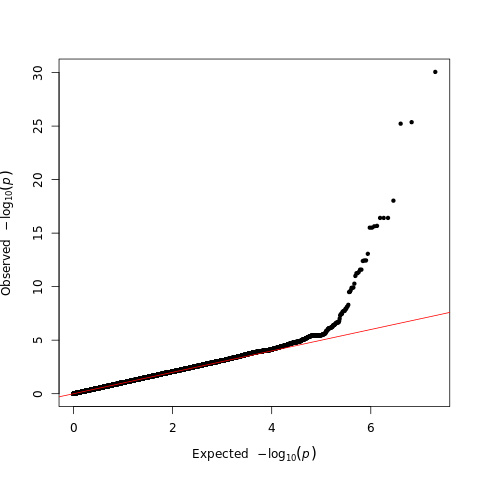


**Supplementary Figure 40: QQ plot of offspring Total Cholesterol.**
